# Lactate-driven pyrimidine synthesis promotes ferroptosis resistance in hepatocellular carcinoma

**DOI:** 10.1186/s43556-026-00450-3

**Published:** 2026-04-17

**Authors:** Mun-Ju Park, Sebin Lee, Dong-Ho Kim, Mi Kyung Kim, Byoung Kuk Jang, Ghilsuk Yoon, Mihyang Park, Gui-Hwa Jeong, Jun-Kyu Byun, Yeon-Kyung Choi, Keun-Gyu Park

**Affiliations:** 1 Department of Biomedical Science, Kyungpook National University, Daegu, 41566 South Korea; 2 Institute of Pharmaceutical Sciences, College of Pharmacy, Kyungpook National University, Daegu, 41566 South Korea; 3 Department of Internal Medicine, Keimyung University School of Medicine, Daegu, 42601 South Korea; 4 Department of Pathology, School of Medicine, Kyungpook National University, Kyungpook National University Chilgok Hospital, Daegu, 41404 South Korea; 5 Research Institute of Aging and Metabolism, Kyungpook National University, Daegu, 41566 South Korea; 6 Department of Internal Medicine, CHA Gumi Medical Center, CHA University, Gumi, 39295 South Korea; 7 Department of Internal Medicine, School of Medicine, Kyungpook National University, Kyungpook National University Chilgok Hospital, Daegu, 41404 South Korea; 8 Department of Internal Medicine, School of Medicine, Kyungpook National University, Kyungpook National University Hospital, Daegu, 41944 South Korea

**Keywords:** Lactate, Hepatocellular carcinoma, Hepatic stellate cells, Pyrimidine biosynthesis, Extracellular matrix, Ferroptosis

## Abstract

Hepatocellular carcinoma (HCC) remains highly lethal, and emerging therapeutic strategies increasingly focus on harnessing ferroptosis to overcome treatment resistance. However, ferroptosis resistance has emerged as a major barrier to these approaches, highlighting the need to identify metabolic cues in the tumor microenvironment that drive this evasion. Here, we identify lactate as a critical metabolite that mediates detrimental metabolic crosstalk between HCC cells and hepatic stellate cells (HSCs), coupling this interaction to pyrimidine biosynthesis and enhanced extracellular matrix (ECM) production within the tumor microenvironment. We show that tumor-derived lactate activates mechanistic target of rapamycin complex 1 (mTORC1)–carbamoyl-phosphate synthetase 2, aspartate transcarbamylase, and dihydroorotase (CAD) signaling, enhancing de novo pyrimidine biosynthesis and pre-ribosomal RNA synthesis, thereby promoting ECM protein translation. The resulting ECM deposition drives Yes-associated protein (YAP)/TEA domain family member (TEAD)-dependent upregulation of the cystine/glutamate antiporter (xCT) in HCC cells, conferring marked resistance to sorafenib-induced ferroptosis. Inhibition of dihydroorotate dehydrogenase, the rate-limiting enzyme in pyrimidine synthesis, disrupts ECM production and restores ferroptosis sensitivity in vitro and in vivo. Clinical data further support these findings, indicating that phosphorylated CAD (p-CAD) levels in HSCs are associated with both poor prognosis and lactate-associated ECM enrichment in HCC patients. Collectively, our study identifies lactate-fueled pyrimidine biosynthesis as a key driver of ECM remodeling and ferroptosis resistance in HCC. Targeting this metabolic axis offers a promising therapeutic strategy to overcome ECM-mediated drug resistance and improve outcomes with ferroptosis-based HCC therapies.

**Supplementary Information:**

The online version contains supplementary material available at 10.1186/s43556-026-00450-3.

## Introduction

Hepatocellular carcinoma (HCC) is a prevalent and lethal liver cancer in which the tumor microenvironment (TME) plays a critical role in disease progression. Within the TME, crosstalk between tumor cells and hepatic stellate cells (HSCs) generates tumor-derived signals that activate HSCs, leading to extracellular matrix (ECM) remodeling that supports tumor growth and invasion [1, 2]. The ECM serves as a structural scaffold integrating cells and tissues and also plays a crucial role in biochemical and biophysical signal transduction [3]. Ferroptosis, an iron-dependent form of regulated cell death driven by lipid peroxidation, has emerged as a promising therapeutic target in cancer [4]. However, the role of ECM accumulation in biophysical signal transduction and its impact on ferroptosis remain unexplored.

Lactate, a major metabolic byproduct of aerobic glycolysis, plays a critical role in shaping the TME. Lactate is transported between cells by monocarboxylate transporters (MCTs) and other related transporters, thereby facilitating intercellular communication and coordination among diverse cells, organs, and tissues [5, 6]. Lactate acts both as a metabolic substrate and as a signaling molecule [7]. In its role as metabolic fuel, lactate is utilized by oxidative tumor cells and stromal cells, promoting metabolic symbiosis within the TME [8]. Lactate functions as a signaling molecule by binding to GPR81 (G protein–coupled receptor 81), Mrs2 (mitochondrial RNA splicing 2), MAVS (mitochondrial antiviral-signaling protein), and NDRG3 (N-myc downstream regulated gene 3), thereby activating relevant signaling pathways [9]. Recent findings suggest that lactate participates in epigenetic regulation through histone lactylation and contributes to HSC activation and liver fibrosis [10]. Despite advances in our understanding of lactate metabolism, its precise role in HSC function, ECM accumulation, and ferroptosis regulation remains inadequately characterized.

Pyrimidine synthesis is essential for DNA replication and cell proliferation, and its dysregulation is a common feature of many cancers [11]. Carbamoyl-phosphate synthetase 2, aspartate transcarbamylase, and dihydroorotase (CAD) and dihydroorotate dehydrogenase (DHODH) are enzymes that work together in the de novo synthesis of pyrimidine [12]. The CAD pathway, comprising carbamoyl-phosphate synthetase 2, aspartate transcarbamylase, and dihydroorotase, is pivotal for pyrimidine synthesis and is tightly regulated by upstream signaling pathways such as mTORC1 (mechanistic target of rapamycin complex 1), MAPK (mitogen-activated protein kinase), and AKT2 (AKT serine/threonine kinase 2) [12, 13]. These pathways modulate CAD activity through phosphorylation, linking pyrimidine synthesis to cellular growth and nutrient availability [12]. DHODH is located on the inner mitochondrial membrane and catalyzes the oxidation of dihydroorotate to orotate, a critical step in pyrimidine biosynthesis [14, 15]. Beyond its role in nucleotide production, DHODH is also involved in mitochondrial bioenergetics, cell proliferation, and reactive oxygen species (ROS) production [15]. Although the role of pyrimidine synthesis in cancer cell proliferation is well-established, its contribution to HSC activation and ECM accumulation remains poorly understood. Understanding how CAD- and DHODH-mediated pyrimidine synthesis affects ECM deposition and ferroptosis susceptibility could provide novel therapeutic insights for HCC treatment.

Here, we hypothesize that cancer-derived lactate stimulates ECM production in HSCs via mTORC1–CAD–DHODH-dependent pyrimidine biosynthesis, thereby promoting ECM accumulation and contributing to ferroptosis resistance in HCC. To test this hypothesis, we dissect the molecular mechanisms linking lactate metabolism to ECM remodeling and ferroptosis escape, and we validate our findings in human HCC tissues. Our study reveals a previously unrecognized metabolic axis linking lactate-fueled pyrimidine biosynthesis to ECM remodeling and ferroptosis resistance, offering both mechanistic insights and novel therapeutic targets for overcoming treatment resistance in HCC.

## Results

### HCC-derived lactate promotes ECM production in HSCs

**Fig. 1:**
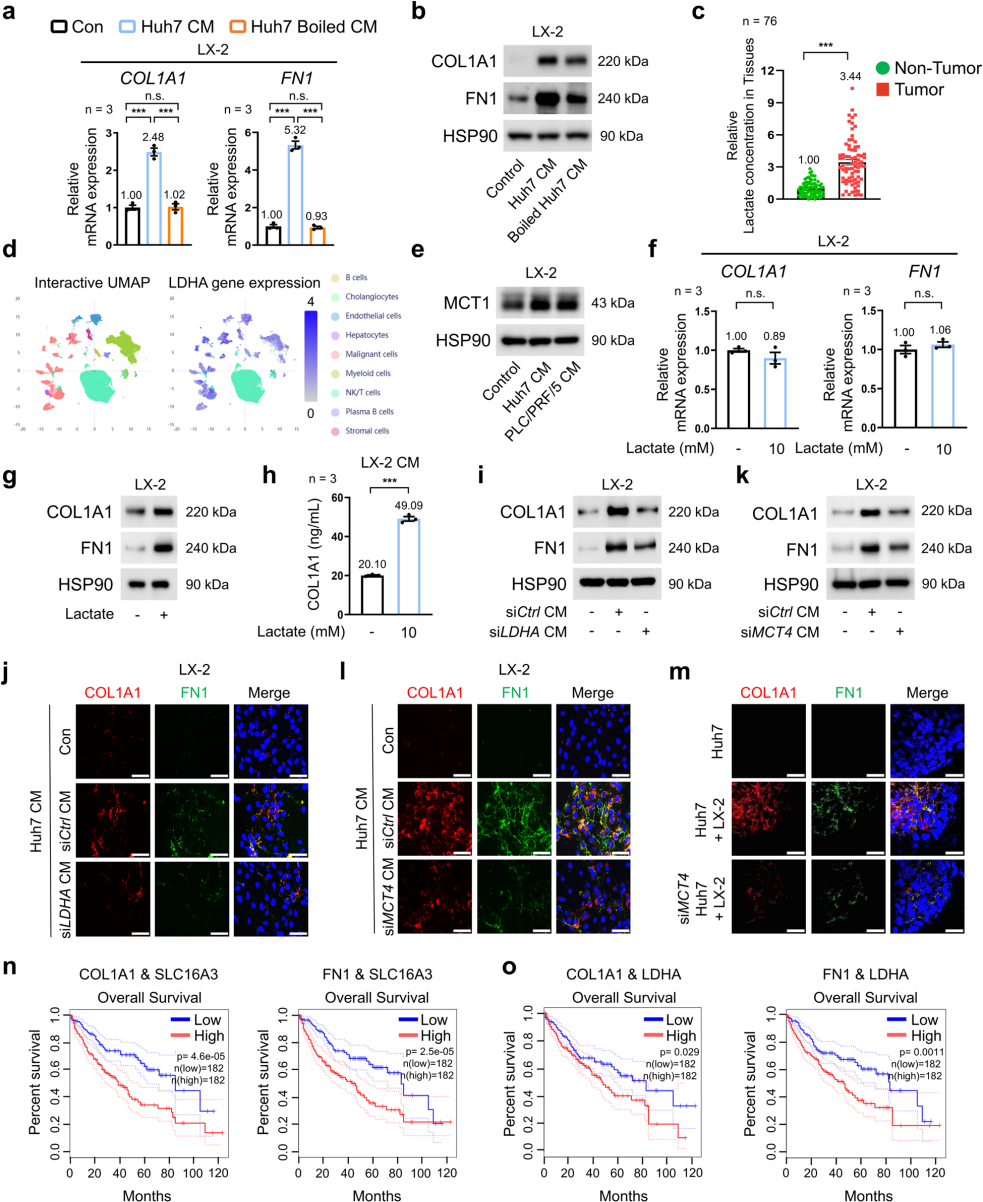
Effects of HCC-derived lactate on ECM production in HSCs. **a**, **b ***COL1A1* and *FN1* mRNA expression (**a**, *n* = 3) and protein levels (**b**) in LX-2 cells cultured for 24 h with conditioned medium (CM) or boiled CM from Huh7 cells. **c** Lactate concentration in tumor tissues and non-tumor tissues from HCC patients (*n* = 76). **d** Single-cell transcriptomic analyses showing *LDHA* gene expression across cancer, immune, and stromal cells in HCC datasets retrieved from the HCCDB (http://lifeome.net/database/hccdb2) **e** MCT1 protein levels in LX-2 cells cultured for 24 h in CM from Huh7 and PLC/PRF/5 cells. **f**, **g ***COL1A1* and *FN1* mRNA expression (**f**, *n* = 3) and protein levels (**g**) in LX-2 cells treated with lactate (10 mM, 24 h). **h** COL1A1 concentrations in CM from LX-2 cells cultured for 24 h in the presence or absence of lactate (10 mM) (*n* = 3). **i**, **j** COL1A1 and FN1 protein levels (**i**) and representative immunofluorescence images of COL1A1 (red) and FN1 (green) (**j**) in LX-2 cells cultured for 24 h with CM from *LDHA*-silenced Huh7 cells. (k, l) COL1A1 and FN1 protein levels (**k**) and representative immunofluorescence images of COL1A1 (red) and FN1 (green) (**l**) in LX-2 cells cultured for 24 h with CM from *MCT4*-silenced Huh7 cells. **m** Representative immunofluorescence images of COL1A1 (red) and FN1 (green) in 3D spheroids: Huh7 alone, Huh7 co-cultured with LX-2, and *MCT4*-silenced Huh7 co-cultured with LX-2. **n**, **o** Kaplan–Meier survival analysis of HCC patients stratified by co-expression levels of COL1A1/SLC16A3 (n, left, *n* = 182/182), FN1/SLC16A3 (n, right, *n* = 182/182), COL1A1/LDHA (**o**, left, *n* = 182/182), and FN1/LDHA (o, right, *n* = 182/182), analyzed using the GEPIA2 database (http://gepia2.cancer-pku.cn). *P* = *p*-value. Fibronectin 1 is referred to as FN1. Scale bars: 75 μm. Data are presented as the mean ± SEM from at least three independent experiments. n.s., not significant; ***p < 0.001

Metabolic factors play an important role in shaping the TME. We therefore examined whether tumor-derived metabolic factors contribute to ECM production in HSCs. To this end, human HSC lines (LX-2) were treated with conditioned media (CM) derived from Huh7 and PLC/PRF/5 cells, which are enriched in bioactive components such as cytokines and metabolites. This treatment significantly increased COL1A1 (collagen type I alpha 1 chain) and fibronectin 1 (FN1) expression at both mRNA and protein levels (Fig. 1a, b; Fig. S1a, b). Boiling the CM, which eliminates the effects of heat-labile cytokines, abolished its ability to induce *COL1A1* and *FN1* mRNA expression, whereas protein levels remained relatively stable (Fig. 1a, b; Fig. S1a, b). Supporting this, active transforming growth factor beta 1 (TGF-β1), a well-known potent inducer of ECM production, was undetectable in both HCC-derived CM and boiled CM, whereas it was readily detected in CM collected from tumor-associated macrophages (TAMs) generated by incubation with HCC-derived CM, as defined by high expression of the TAM markers *CD206* (cluster of differentiation 206) and *CD163* (Fig. S1c, d). This finding suggests that CM-derived heat-stable metabolites, such as lactate, play a role in tumor ECM deposition by regulating ECM production at the translational level, potentially indicating that they exert a greater effect on shaping the TME than heat-labile factors. Supporting this notion, lactate levels were significantly higher in human HCC tissues than in adjacent non-tumor tissues, suggesting that dysregulatedlactate metabolism may be involved in the pathophysiology of HCC (Fig. 1c). Furthermore, single-cell transcriptomic analyses of publicly available HCC datasets using the HCCDB database showed that lactate dehydrogenase A (LDHA) (which catalyzes the conversion of pyruvate to lactate as the final step of glycolysis) was more highly expressed in cancer cells than in stromal or immune cells (Fig. 1d), consistent with prior reports that lactate-related gene signatures are enriched in malignant cancer cells [16]. Moreover, consistent with this metabolic shift and prior observations [17], exposure of LX-2 cells to cancer-derived CM markedly upregulated MCT1, a key regulator of lactate flux (Fig. 1e). To confirm lactate as a critical metabolite in CM-induced ECM regulation, LX-2 cells were treated with lactate at concentrations equivalent to those in HCC-derived CM and boiled CM (~10 mM; Fig. S1e). This concentration, comparable to lactate levels observed in tumor tissue (10–30 mM) [18], increased ECM protein levels, whereas mRNA expression remained unchanged, mirroring the effects of boiled CM (Fig. 1f, g). Consistently, CM derived from lactate-treated LX-2 cells exhibited increased levels of secreted COL1A1 proteins, further supporting lactate-dependent enhancement of ECM production (Fig. 1h). The CM-induced upregulation of ECM proteins in LX-2 cells was abolished by silencing *LDHA* (Fig. 1i; Fig. S1f, g). This result was confirmed by immunofluorescence analysis (Fig. 1j; Fig. S1h). To confirm the role of lactate transport, LX-2 cells were treated with CM from *MCT4*-silenced HCC cells, which resulted in a similar reduction in ECM proteins (Fig. 1k, l; Fig. S1i–k). Taken together, these results indicate that HCC cell-derived lactate serves as a pivotal metabolic driver of ECM production in HSCs. The functional consequences of ECM deposition were assessed using a co-culture spheroid system comprising LX-2 and HCC cells. *MCT4* silencing in HCC cells led to reduced ECM protein deposition (Fig. 1m; Fig. S1l). To determine the clinical relevance of lactate-associated ECM production, we analyzed gene expression profiles and overall survival using publicly available HCC datasets. *COL1A1* and *FN1* mRNA levels were markedly higher in tumor tissues than in adjacent normal tissues, as assessed using the TNMplot web tool (Fig. S1m). However, although the transcriptional upregulation of either gene alone did not significantly correlate with patient prognosis, co-expression with elevated *LDHA* or *SLC16A3* (solute carrier family 16 member 3) levels was associated with reduced overall survival, based on GEPIA2 (Gene Expression Profiling Interactive Analysis 2) survival analysis, thereby supporting a link between lactate-driven enhancement of ECM protein abundance and adverse clinical outcomes in HCC (Fig. 1n, o; Fig. S1n). Collectively, these findings suggest that cancer-derived lactate functions as a metabolic signal that activates HSCs and drives ECM remodeling, thereby potentially contributing to adverse clinical outcomes in HCC.

### The mTORC1-dependent CAD pathway drives lactate-induced ECM production in HSCs

**Fig. 2:**
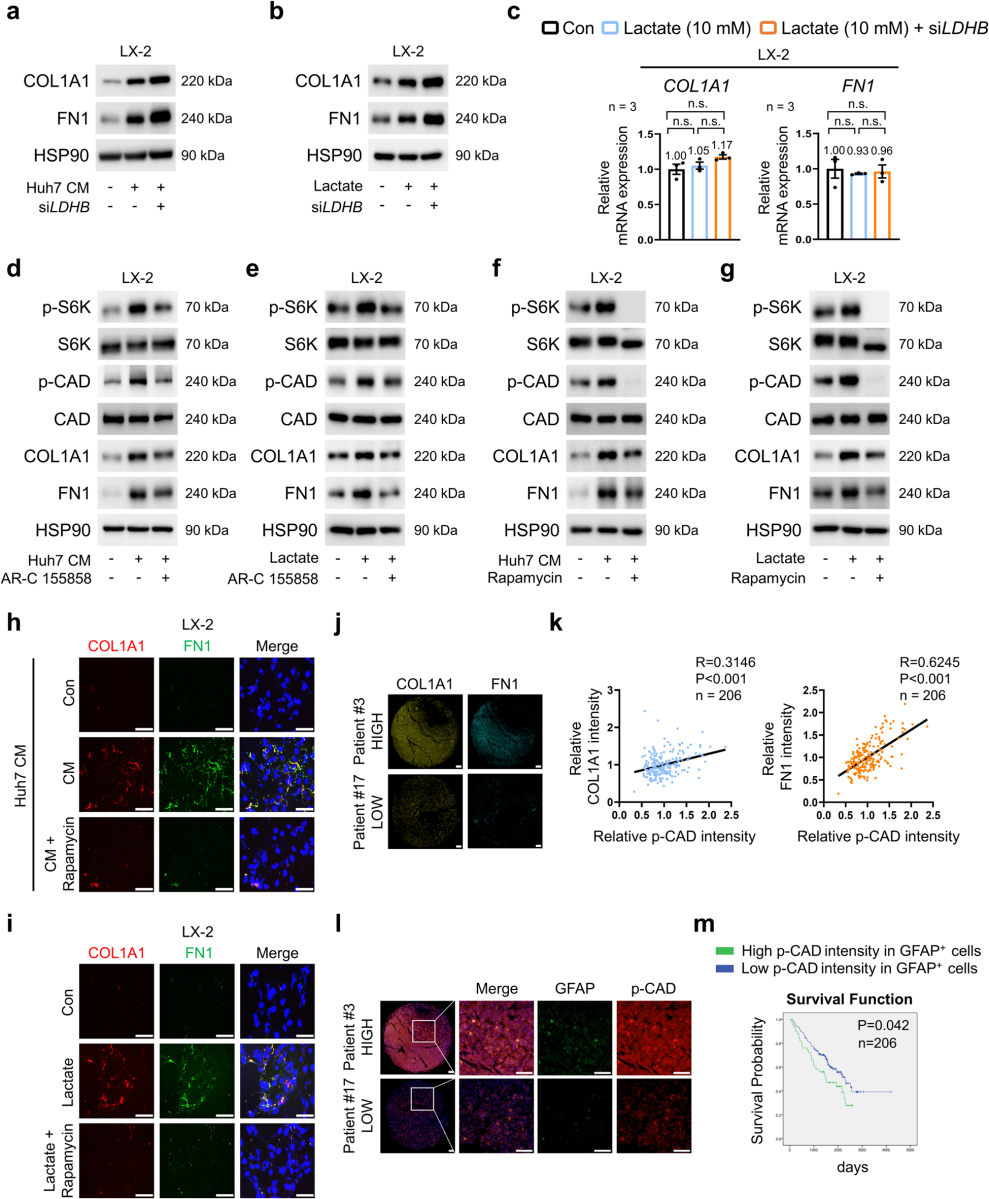
Effects of lactate on the mTORC1-CAD pathway and ECM production in HSCs. **a**, **b** COL1A1 and FN1 protein levels in *LDHB*-silenced LX-2 cells cultured in CM from Huh7 cells (**a**) or in the presence or absence of lactate (10 mM, 24 h) (**b**). **c** Relative mRNA expression of *COL1A*1 and *FN1* in *LDHB*-silenced LX-2 cells cultured in the presence or absence of lactate (10 mM, 24 h) (*n* = 3). **d**, **e** Effects of AR-C155858 (2 µM, 24 h) on phosphorylated S6K, CAD, COL1A1, and FN1 levels in LX-2 cells cultured in CM from Huh7 cells (**d**) or in the presence or absence of lactate (10 mM, 24 h) (**e**). **f**, **g** Effects of rapamycin (20 nM, 24 h) on phosphorylated S6K, CAD, COL1A1, and FN1 levels in LX-2 cells cultured in CM from Huh7 cells (**f**) or in the presence or absence of lactate (10 mM, 24 h) (g). **h**, **i** Representative immunofluorescence images of COL1A1 (red) and FN1 (green) in LX-2 cells showing the effects of rapamycin (20 nM, 24 h) in CM from Huh7 cells (**h**) or in the presence or absence of lactate (10 mM, 24 h) (**i**). **j** Tissue microarray analysis of COL1A1 and FN1 expression in HCC tumor tissues categorized into high- and low-expression groups (*n* = 206; high expression: *n* = 50, low expression: *n* = 156). **k** Correlation between phosphorylated CAD intensity and COL1A1 intensity (left) or FN1 intensity (right) in the HCC tissue microarray (*n* = 206). R = Pearson correlation coefficient; *P* = *p*-value. **l** Representative tissue microarray images of GFAP and phosphorylated CAD expression in HCC tumor tissues from patients with high or low expression (*n* = 206). **m** Kaplan–Meier survival curve comparing high and low phosphorylated CAD intensity in GFAP-positive cells in HCC tissue microarray samples (*n* = 206; high intensity: *n* = 50, low intensity: *n* = 156). *P* = *p*-value. Scale bar: 75 µm. Data are presented as the mean ± SEM from at least three independent experiments. N.s., not significant

To determine the molecular mechanisms by which lactate regulates ECM production in HSCs, we first silenced *LDHB*, which catalyzes the oxidation of lactate to pyruvate and supports lactate utilization [19]. Intriguingly, silencing *LDHB* further augmented the CM- or lactate-induced upregulation of COL1A1 and FN1 protein levels without affecting mRNA expression (Fig. 2a–c; Fig. S2a–d). This paradoxical enhancement indicates that intracellular lactate accumulation, rather than metabolic conversion, contributes to ECM protein production in HSCs. Next, we explored how accumulated lactate enhances ECM protein synthesis, focusing on the mTORC1 signaling pathway, a central regulator of protein translation. Given prior evidence linking lactate to mTORC1 activation [20–23] and mTORC1’s role in promoting pyrimidine biosynthesis through CAD activation [24], we explored the effect of lactate-induced mTORC1 activation and subsequent CAD phosphorylation on ECM production. Treatment with HCC-derived CM or lactate increased mTORC1 activity, as indicated by phosphorylation of S6K (ribosomal protein S6 kinase [T389]), and CAD phosphorylation, alongside increased levels of COL1A1 and FN1 in LX-2 cells (Fig. 2d, e; Fig. S2e). These effects were significantly decreased by AR-C155858, an MCT1 inhibitor, which reduced COL1A1 and FN1 protein levels without affecting mRNA expression (Fig. 2d, e; Fig. S2e–h). These responses were recapitulated in primary hepatic stellate cells (pHSCs), which were validated by *high cytoglobin* (*Cygb*) and *glial fibrillary acidic protein* (*Gfap*) expression and negligible *Albumin* levels (Fig. S2i-k). Notably, silencing the lactate receptor *GPR81* did not alter mTORC1 activity, CAD phosphorylation, or the protein levels of COL1A1 and FN1 (Fig. S2l, m), indicating that lactate’s effects are primarily mediated by MCT1-dependent lactate uptake. Beyond its role in mTORC1 activation, lactate has been reported to regulate cellular responses through HIF-1α signaling and histone lactylation [25, 26]. To investigate the potential involvement of these pathways, we assessed their contribution to lactate-induced ECM production in HSCs. AR-C155858 treatment did not induce significant changes in HIF-1α expression or histone H3 lysine 18 lactylation (H3K18la) (Fig. S2n). Furthermore, neither silencing of *HIF-1α* nor *EP300*, a key histone acetyltransferase implicated in histone lactylation [27], altered S6K phosphorylation, CAD phosphorylation, or ECM protein levels (Fig. S2o, p), supporting the specificity of the mTORC1-CAD axis in this process. Treatment with rapamycin, an mTORC1 inhibitor, suppressed CM- or lactate-induced phosphorylation of S6K and CAD, concomitant with decreased ECM production in LX-2 cells (Fig. 2f–i; Fig. S3a, b). By contrast, silencing *LDHB* upregulated mTORC1-dependent CAD phosphorylation signaling (Fig. S3c), confirming the effects of lactate levels on mTORC1-dependent CAD phosphorylation. Lactate-driven ECM production was abolished when *RPTOR*, but not *RICTOR*, was silenced in LX-2 cells, highlighting the mTORC1 dependency of this process (Fig. S3d, e). The loss of lactate-induced ECM production in *S6K*- or *CAD*-silenced LX-2 cells further validated the critical role of the mTORC1-CAD signaling axis in mediating lactate-driven ECM synthesis (Fig. S3f, g). Since mTORC1 also coordinates translation initiation via the 4E-BP1–eIF4E axis, we next evaluated the relative contribution of this branch to lactate-driven ECM synthesis [28]. Although lactate also activated the 4E-BP1–eIF4E axis, *CAD* inhibition more potently reduced ECM production than *eIF4E* suppression, and combined inhibition did not produce additive effects (Fig. S3h–j). In our patient cohort, we examined the regulatory effect of phosphorylated CAD (p-CAD) on ECM expression by analyzing a tumor microarray of 206 patients with HCC who underwent liver resection (Table S1). The intensity of COL1A1 and FN1 showed a positive correlation with the intensity of p-CAD (Fig. 2j, k). Higher p-CAD intensity in GFAP (an early marker of HSC activation)-positive HSCs was associated with poorer survival outcomes (Fig. 2l, m), underscoring the clinical relevance of CAD-mediated pyrimidine biosynthesis in HCC progression. These results suggest that mTORC1–CAD-dependent pyrimidine biosynthesis may serve as a key mechanism through which lactate enhances ECM production in HSCs and indicate its association with poor prognosis in HCC.

### Inhibition of CAD and DHODH impairs lactate-driven ECM production in HSCs

**Fig. 3:**
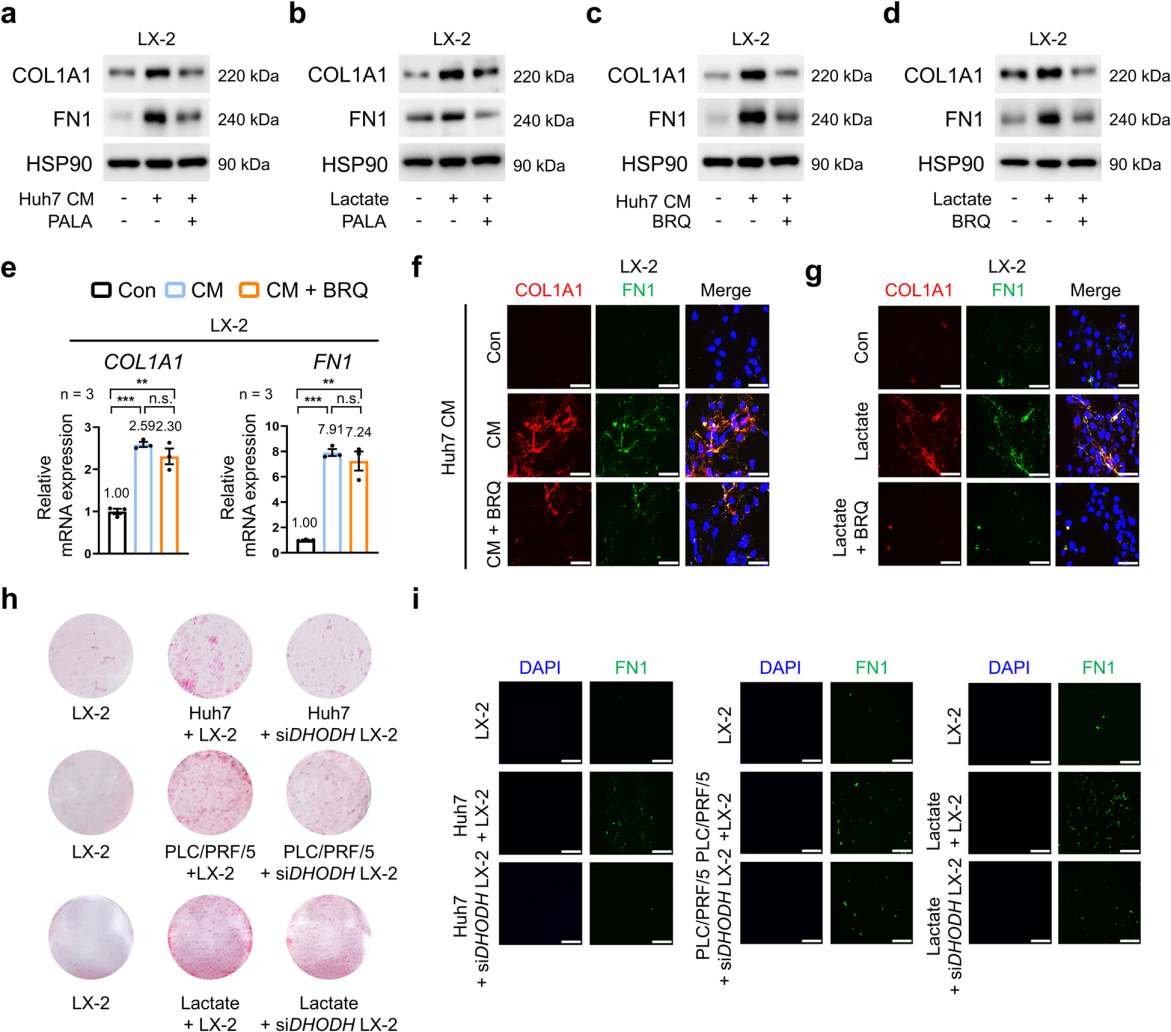
Effects of pyrimidine biosynthesis on lactate-induced ECM production in HSCs. **a**, **b** Effects of PALA (150 µM, 24 h) on COL1A1 and FN1 protein levels in LX-2 cells cultured in CM from Huh7 cells (**a**) or in the presence or absence of lactate (10 mM, 24 h) (**b**). **c**, **d** Effects of BRQ (0.1 µM, 24 h) on COL1A1 and FN1 protein levels in LX-2 cells cultured in CM from Huh7 cells (c) or in the presence or absence of lactate (10 mM, 24 h) (**d**). **e** Effects of BRQ (0.1 µM, 24 h) on *COL1A1* and *FN1* mRNA expression in LX-2 cells cultured in CM from Huh7 cells (*n* = 3). **f**, **g** Representative immunofluorescence images of COL1A1 (red) and FN1 (green) in LX-2 cells showing the effects of BRQ (0.1 µM, 24 h) in CM from Huh7 cells (**f**) or in the presence or absence of lactate (10 mM, 24 h) (**g**). **h** Representative picrosirius red staining showing collagen deposition in Huh7 or PLC/PRF/5 cells co-cultured with *DHODH*-silenced LX-2 cells in the presence or absence of lactate (10 mM, 24 h). **i** Representative images showing immunofluorescence staining of FN1 in Huh7 or PLC/PRF/5 cells co-cultured with *DHODH*-silenced LX-2 cells in the presence or absence of lactate (10 mM, 24 h). Scale bar: 75 µm. Data are presented as the mean ± SEM from at least three independent experiments. n.s., Not significant; **p < 0.01; ***p < 0.001. PALA, N-phosphonacetyl-L-aspartate; BRQ, brequinar

To further validate the functional requirement of pyrimidine biosynthesis in lactate-induced ECM production in HSCs, we examined whether inhibition of pyrimidine biosynthesis could mitigate ECM production in HSCs stimulated with HCC-derived CM or lactate. Treatment with PALA (N-phosphonacetyl-L-aspartate), an inhibitor targeting the aspartate transcarbamylase activity of CAD, significantly reduced COL1A1 and FN1 protein levels in LX-2 cells stimulated with HCC-derived CM or lactate (Fig. 3a, b; Fig. S4a). PALA selectively inhibits ATCase activity within CAD and thus may not fully block pyrimidine biosynthesis, as downstream enzymatic activity remains intact [29]. DHODH is a key enzyme in pyrimidine synthesis that catalyzes the oxidation of dihydroorotate to orotate, a precursor for uridine monophosphate (UMP) and all pyrimidine nucleotides; inhibition of DHODH suppresses pyrimidine biosynthesis, promotes immune responses, and has anticancer effects [13, 30, 31]. We therefore hypothesized that disrupting DHODH could provide further insight into the role of pyrimidine metabolism in ECM regulation in HSCs [12, 32]. Consistent with the effects observed with the CAD inhibitor, treatment with brequinar (BRQ), a DHODH inhibitor, significantly decreased COL1A1 and FN1 protein levels elevated by HCC-derived CM or lactate stimulation without affecting mRNA levels**,** thus implicating pyrimidine biosynthesis in the post-transcriptional regulation of ECM components in LX-2 cells and primary HSCs (Fig. 3c–e; Fig. S4b–f). Immunofluorescence staining showed that BRQ treatment decreased the accumulation of COL1A1 and FN1 induced by HCC-derived CM or lactate (Fig. 3f, g; Fig. S4g). To extend these findings, we examined ECM deposition using Picro Sirius staining and a two-dimensional co-culture system comprising LX-2 cells and HCC cells, which showed reduced ECM production following *DHODH* silencing in LX-2 cells (Fig. 3h, i; Fig. S4h). Collectively, these findings suggest that pyrimidine biosynthesis via the CAD and DHODH pathway is essential for lactate-driven ECM production in HSCs, further pointing to the presence of a metabolic axis linking pyrimidine metabolism to ECM remodeling in the TME.

###  Lactate-driven pyrimidine biosynthesis promotes ECM production via pre-ribosomal RNA (rRNA)-dependent translation in HSCs

**Fig. 4:**
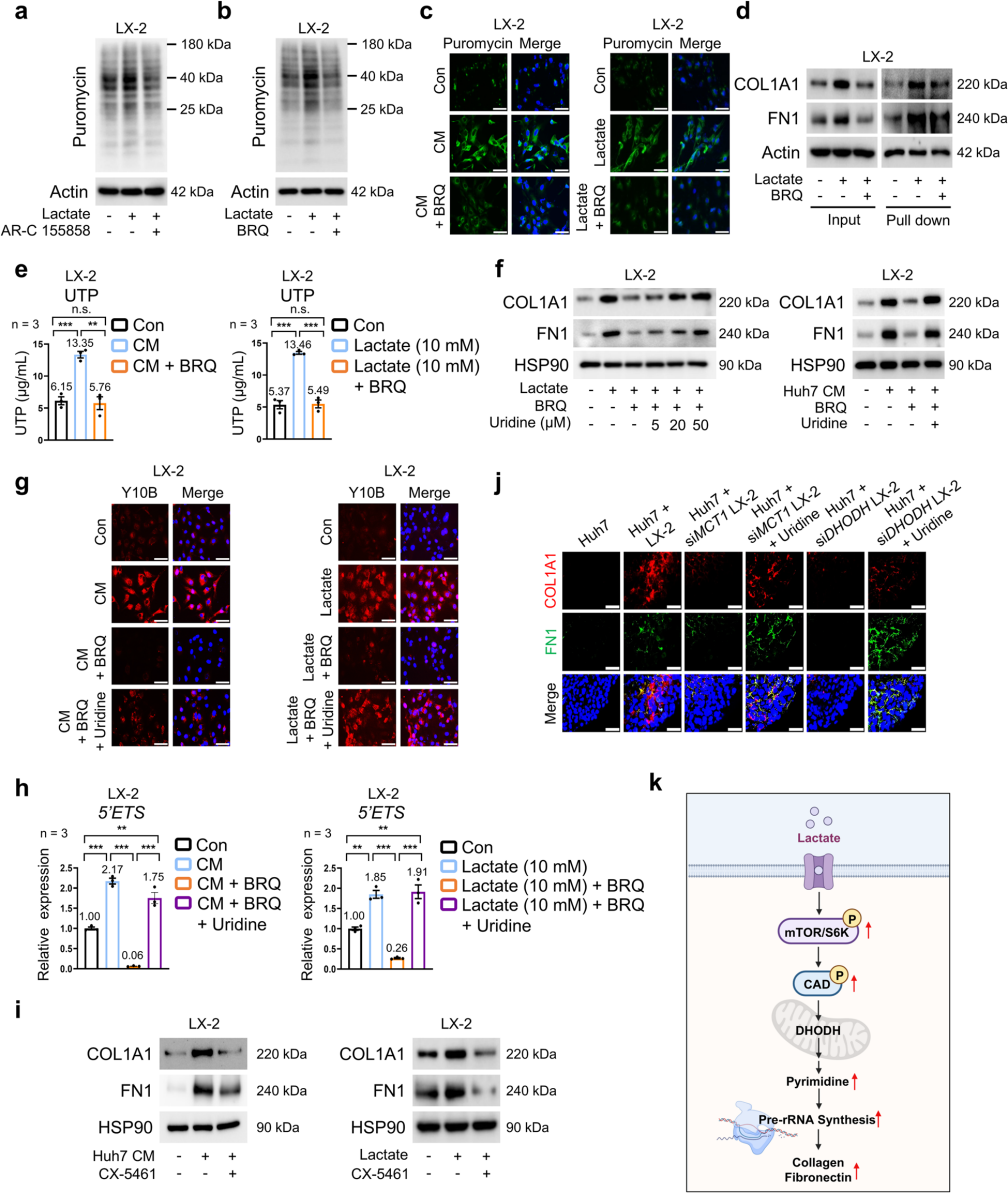
Effects of pyrimidine biosynthesis-driven pre-rRNA synthesis on lactate-induced ECM production. **a**, **b** Effects of 24 h exposure to AR-C155858 (2 µM, a) or BRQ (0.1 µM, b) on puromycin incorporation in LX-2 cells cultured in the presence or absence of lactate (10 mM). **c** Immunofluorescence staining for puromycin (1 µg/mL) in LX-2 cells showing the effects of BRQ (0.1 µM, 24 h) in CM from Huh7 cells (left) or in the presence or absence of lactate (10 mM, 24 h, right). **d** Pull-down assay showing the effects of BRQ (0.1 µM, 24 h) on COL1A1 and FN1 protein levels in LX-2 cells cultured in the presence or absence of lactate (10 mM, 24 h). **e** UTP levels in LX-2 cells treated with BRQ (0.1 µM, 24 h) in CM from Huh7 cells (left) or in the presence or absence of lactate (10 mM, 24 h, right) (*n* = 3). **f** Effects of BRQ or uridine on COL1A1 and FN1 protein levels in LX-2 cells. LX-2 cells were treated with BRQ (0.1 µM) or uridine (5, 20, or 50 µM) in the absence or presence of lactate (10 mM) for 24 h (left panel). LX-2 cells cultured in Huh7-derived CM were treated with BRQ (0.1 µM) in the absence or presence of uridine (50 µM) for 24 h (right panel). **g** Immunofluorescence staining of Y10B in LX-2 cells showing the effects of BRQ (0.1 µM) or uridine (50 µM) exposure for 24 h in CM from Huh7 cells (left) or in the presence or absence of lactate (10 mM, right). **h** Relative expression of 5′*ETS* in LX-2 cells treated with BRQ (0.1 µM) or uridine (50 µM) for 24 h in CM from Huh7 cells (left) or in the presence or absence of lactate (10 mM, right) (*n* = 3). **i** Effects of CX-5461 (0.1 µM, 24 h) on COL1A1 and FN1 protein levels in LX-2 cells cultured in CM from Huh7 cells (left) or in the presence or absence of lactate (10 mM, 24 h, right). **j** Effects of *MCT1*- or *DHODH*-targeting siRNA on COL1A1 (red) and FN1 (green) immunofluorescence staining in LX-2 cells co-cultured with Huh7 cells and treated with uridine (50 µM, 24 h). **k** Schematic model illustrating lactate-driven mTORC1 activation and its role in ECM production via the pre-rRNA synthesis pathway in HSCs. Created with BioRender.com, agreement number: MP29JVSCPU. Scale bar: 75 µm. Data are presented as the mean ± SEM from at least three independent experiments. n.s., Not significant; **p < 0.01; ***p < 0.001. BRQ, Brequinar

Next, we explored the mechanism by which pyrimidine biosynthesis mediates ECM production in HCC-derived CM- or lactate-treated HSCs. DHODH, located in the mitochondrial inner membrane, plays a pivotal role in cellular metabolism by coupling the oxidation of dihydroorotate to orotate with the reduction of CoQ (coenzyme Q) to its reduced form (CoQH_2_) [12]. To determine whether BRQ-mediated DHODH inhibition affects ECM protein levels by inducing mitochondrial dysfunction, we evaluated the oxygen consumption rate (OCR) in lactate-treated LX-2 cells. BRQ treatment decreased lactate-induced OCR (Fig. S5a); however, supplementation with MitoQH_2_, a mitochondria-targeted analog of CoQH_2_, failed to reverse BRQ-induced suppression of COL1A1 and FN1 in LX-2 cells (Fig. S5b). Similar findings were obtained in LX-2 cells treated with HCC-derived CM (Fig. S5c, d), indicating that the effect of DHODH inhibition on ECM production is not mediated by mitochondrial dysfunction. Given the established link between the de novo pyrimidine synthesis pathway, pre-rRNA (precursor rRNA) synthesis, and protein synthesis [33], we next investigated the involvement of pre-rRNA in pyrimidine synthesis-mediated ECM production in HSCs. Pre-rRNA synthesis is essential for ribosomal biogenesis by facilitating the formation of ribosomes that translate mRNA into proteins required for cellular growth and metabolism [34]. To determine whether HSC activation by HCC-derived CM or lactate involves translational regulation, we monitored protein synthesis using puromycin labeling. Both AR-C155858 and BRQ treatments reduced puromycin incorporation (Fig. 4a, b; Fig. S5e, f). Immunofluorescence analysis showed reduced levels of puromycin-labeled proteins, indicating suppression of protein synthesis upon DHODH inhibition (Fig. 4c; Fig. S5g). Consistently, BRQ-mediated DHODH inhibition decreased nascent COL1A1 and FN1 protein levels, as demonstrated by L-azidohomoalanine (AHA) labeling, which tracks newly synthesized proteins (Fig. 4d; Fig. S5h). Consistent with the role of DHODH in UTP (uridine triphosphate) production, UTP levels increased in response to HCC-derived CM or lactate treatment, whereas they decreased upon BRQ treatment (Fig. 4e; Fig. S5i). Uridine supplementation rescued the BRQ-mediated suppression of COL1A1 and FN1 protein levels. To define the effective concentration required for this recovery, we performed a dose–response analysis using 5, 20, and 50 μM uridine. Treatment with 5 or 20 μM uridine resulted in incomplete restoration of ECM protein expression, whereas 50 μM uridine induced a near-complete recovery of both COL1A1 and FN1 levels (Fig. 4f; Fig. S5j), supporting the use of 50 μM uridine for subsequent experiments. To determine whether this rescue effect was specific to uridine, we next tested cytidine—another nucleoside generated through de novo pyrimidine biosynthesis—and found that it similarly restored BRQ-induced reductions in COL1A1 and FN1 protein levels (Fig. S5k). Uridine supplementation also restored pre-rRNA synthesis, as indicated by recovery of Y10B anti-rRNA monoclonal antibody expression, a marker of ribosome biogenesis (Fig. 4g; Fig. S5l). Pre-rRNA is relatively unstable and rapidly undergoes post-transcriptional processing and maturation; therefore, the 5′ externally transcribed spacer (ETS) region is a reliable indicator of active pre-rRNA synthesis and provides information on ongoing rRNA production [35]. Treatment with HCC-derived CM or lactate upregulated *5′ETS* region expression, which was attenuated by BRQ treatment and rescued by uridine supplementation, further supporting the restoration of pre-rRNA synthesis (Fig. 4h; Fig. S5m). To explore the causal link between rRNA synthesis and ECM regulation, we used CX-5461, an RNA polymerase I inhibitor that suppresses rRNA synthesis. CX-5461 treatment significantly reduced ECM protein levels induced by HCC-derived CM or lactate (Fig. 4i; Fig. S5n). Finally, immunofluorescence analysis showed that silencing *MCT1* or *DHODH* in LX-2 cells co-cultured with HCC cells reduced COL1A1 and FN1 levels, which were restored upon uridine supplementation (Fig. 4j; Fig. S5o, p). Collectively, these findings indicate that lactate-driven pyrimidine biosynthesis promotes ECM production in HSCs by inducing pre-rRNA-dependent translation (Fig. 4k).

### Collagen-DDR1-YAP signaling drives ferroptosis resistance in HCC, and targeting DHODH in HSCs restores ferroptosis sensitivity

**Fig. 5:**
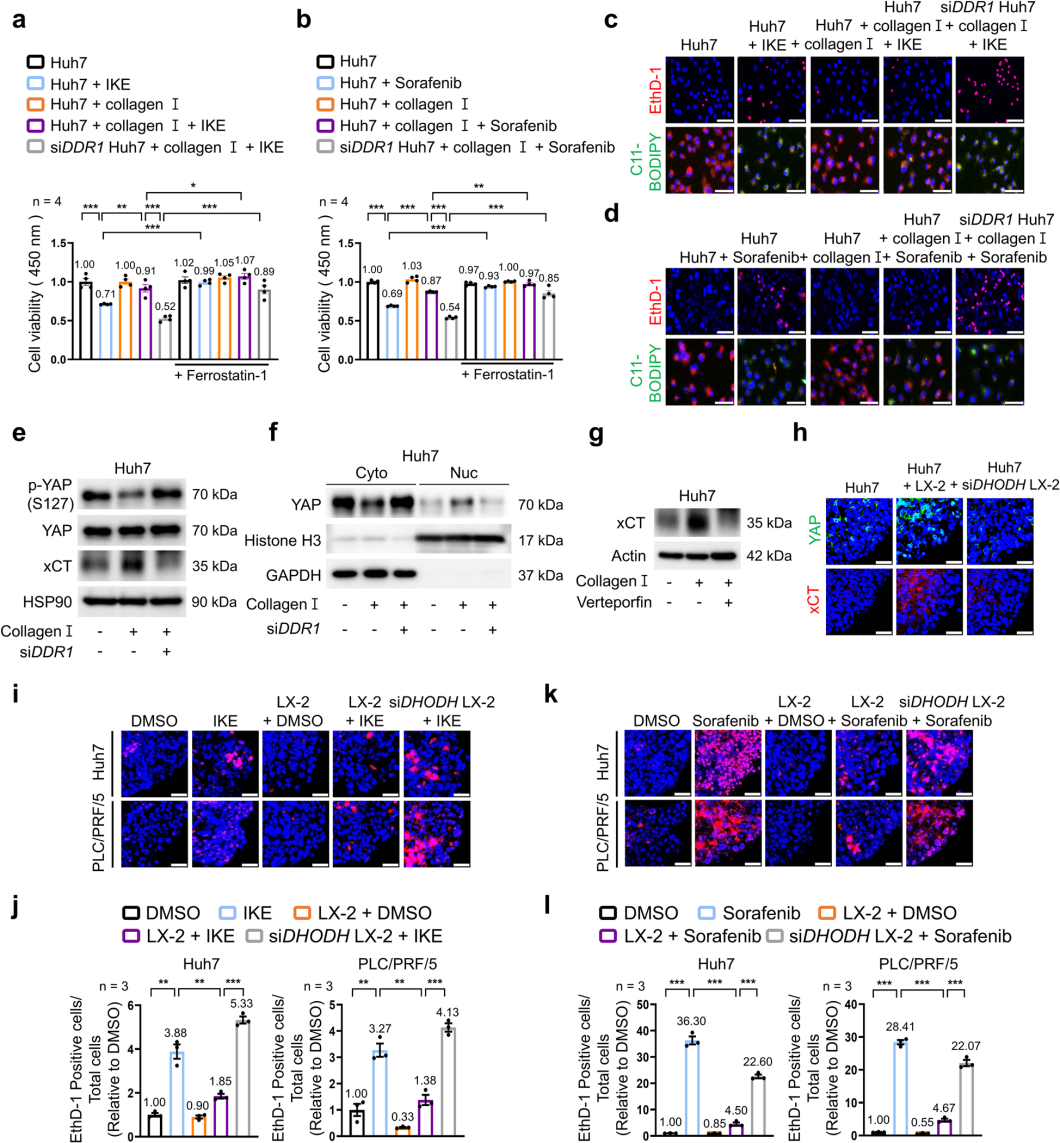
Effects of collagen-driven YAP activation on resistance to sorafenib-induced ferroptosis in HCC. **a** Cell viability measured by CCK-8 assay in Huh7 cells treated with IKE (1 µM, 6 h) in the presence or absence of collagen I (30 µg/mL, 24 h), ferrostatin-1 (1 µM, 24 h), or *DDR1*-targeting siRNA (*n* = 4). **b** Cell viability measured by CCK-8 assay in Huh7 cells treated with sorafenib (10 µM, 6 h) in the presence or absence of collagen I (30 µg/mL, 24 h), ferrostatin-1 (1 µM, 24 h), or *DDR1*-targeting siRNA (*n* = 4). **c** Representative images of EthD-1 (red, 4 µM) and C11-BODIPY (green, 10 µM) staining in Huh7 cells treated with IKE (1 µM, 6 h) in the presence or absence of collagen I (30 µg/mL, 24 h) or *DDR1*-targeting siRNA. **d** Representative images of EthD-1 (red, 4 µM) and C11-BODIPY (green, 10 µM) staining in Huh7 cells treated with sorafenib (10 µM, 6 h) in the presence or absence of collagen I (30 µg/mL, 24 h) or *DDR1*-targeting siRNA. **e** Protein levels of phosphorylated YAP (S127) and xCT in *DDR1*-silenced Huh7 cells in the presence or absence of collagen I (30 µg/mL, 24 h). **f** Protein levels of YAP in cytoplasmic and nuclear fractions of *DDR1*-silenced Huh7 cells in the presence or absence of collagen I (30 µg/mL, 24 h). Histone H3 and GAPDH were used as nuclear and cytoplasmic markers, respectively. **g** Protein levels of xCT in Huh7 cells treated with collagen I (30 µg/mL, 24 h) or verteporfin (1 µM, 24 h). **h** Representative immunofluorescence images of YAP and xCT in *DHODH*-silenced LX-2 cells co-cultured with Huh7 cells. **i**, **j** Representative images (**i**) and quantification (**j**) of EthD-1 (red, 4 µM) staining in *DHODH*-silenced LX-2 cells co-cultured with Huh7 and PLC/PRF/5 cells and treated with IKE (1 µM, 6 h) (*n* = 3). (**k**, **l**) Representative images (**k**) and quantification (**l**) of EthD-1 (red, 4 µM) staining in *DHODH*-silenced LX-2 cells co-cultured with Huh7 and PLC/PRF/5 cells and treated with sorafenib (10 µM, 6 h) (*n* = 3). Scale bar: 75 µm. Data are presented as the mean ± SEM from at least three independent experiments. *p < 0.05; **p < 0.01; ***p < 0.001. PLC, PLC/PRF/5; IKE, imidazole-ketone-erastin

Accumulating evidence indicates a strong association between altered ferroptosis sensitivity and drug resistance in HCC [36, 37], but the impact of ECM production on ferroptosis resistance remains largely unexplored. On the basis of our earlier findings that HCC-derived CM or lactate stimulates COL1A1 production in HSCs, we tested whether this collagen-rich ECM directly contributes to ferroptosis resistance in HCC cells. To distinguish the effects of ECM components from other CM-derived factors, we used purified collagen to functionally isolate the contribution of COL1A1 itself. We confirmed that cell death induced by sorafenib or IKE (imidazole ketone erastin) reflected ferroptosis, as ferrostatin-1 restored cell viability (Fig. 5a, b; Fig. S6a, b). Collagen treatment attenuated IKE- or sorafenib-induced ferroptosis and lipid ROS accumulation, as evidenced by ethidium homodimer-1 (EthD-1) and C11-BODIPY staining (Fig. 5c, d; Fig. S6c–h). Given a previous study showing that COL1A1 and its receptor discoidin domain receptor 1 (DDR1) induce YAP (Yes-associated protein) nuclear translocation in HCC cells [38], we hypothesized that collagen-induced YAP activation is essential for ferroptosis resistance. In line with this hypothesis, fibronectin did not activate YAP, indicating that YAP signaling is driven by collagen (Fig. S7a, b). Moreover, *DDR1* silencing not only reversed ferroptosis resistance—whereas treatment with the apoptosis inhibitor Z-VAD-FMK or the necroptosis inhibitor necrostatin-1 failed to rescue cell viability (Fig. 5a–d; Fig. S7c-h)—but also attenuated collagen-stimulated YAP activation (Fig. 5e). Specifically, *DDR1* knockdown significantly reduced nuclear YAP accumulation and diminished the collagen-induced upregulation of the cystine/glutamate antiporter xCT (SLC7A11 [solute carrier family 7 member 11]) at both mRNA and protein levels (Fig. 5e, f; Fig. S8a-d). TEAD (TEA domain) reporter assays further confirmed that collagen-induced TEAD activity was abolished by *DDR1* knockdown, supporting the involvement of the COL1A1–DDR1–YAP axis in ferroptosis resistance (Fig. S8e, f). Further delineating this mechanism, the YAP inhibitor verteporfin suppressed the collagen-induced upregulation of *xCT* mRNA and protein levels but did not similarly alter the protein levels of the key ferroptosis regulators GPX4 (glutathione peroxidase 4) or ACSL4 (acyl-CoA synthetase long-chain family member 4) (Fig. 5g; Fig. S8g–k). Likewise, the TEAD inhibitor MGH-CP1 similarly suppressed collagen-induced *xCT* mRNA expression (Fig. S8l, m), thereby positioning the YAP-TEAD axis as a specific transcriptional driver of xCT expression. Finally, in an LX-2/HCC spheroid co-culture system, LX-2 cells enhanced YAP nuclear translocation and increased xCT expression in HCC cells, whereas co-culture with *DHODH*-silenced LX-2 cells abrogated these effects (Fig. 5h; Fig. S8n). Co-culture with LX-2 cells also inhibited IKE- or sorafenib-induced ferroptosis in HCC cells, and this ferroptosis resistance was reversed by silencing *DHODH* in LX-2 cells (Fig. 5i–l). Together, these findings indicate that ECM components contribute to ferroptosis resistance in HCC cells and support targeting DHODH in LX-2 cells as a feasible approach to enhance the response to sorafenib by promoting ferroptosis.

### Targeting tumor-derived lactate and pyrimidine biosynthesis in HSCs increases sorafenib efficacy in HCC

**Fig. 6:**
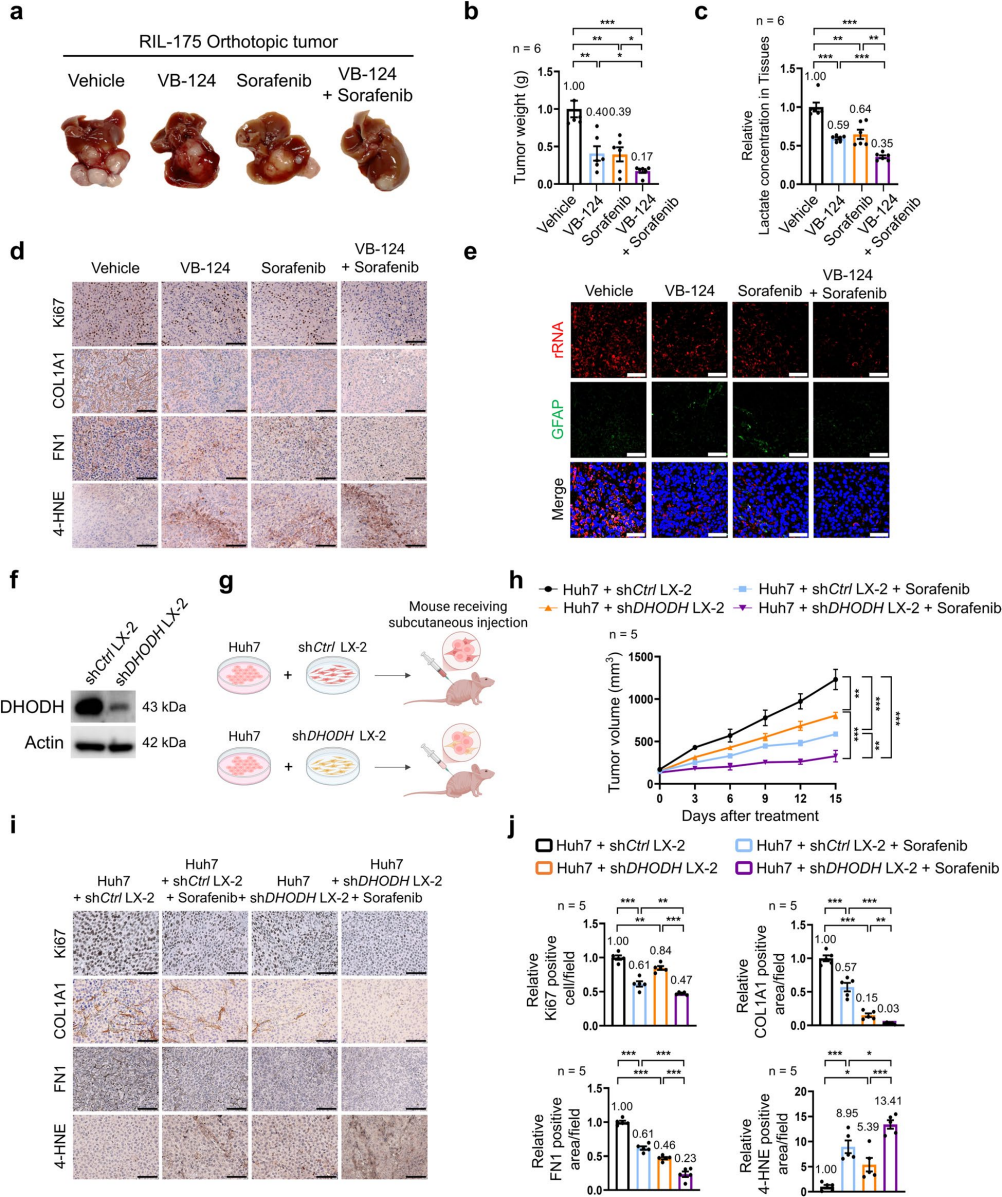
Effects of targeting tumor-derived lactate or pyrimidine biosynthesis on tumor growth. **a**–**c** Representative gross images (**a**), tumor weight (**b**), and relative lactate concentrations in tumor tissues (**c**) from orthotopic RIL-175 tumors in C57BL/6 mice treated with VB-124 (10 mg/kg) or sorafenib (5 mg/kg) (*n* = 6 per group). **d** Immunohistochemical staining for Ki67, COL1A1, FN1, and 4-HNE in RIL-175 orthotopic tumor tissues. **e** Representative immunofluorescence images of rRNA and GFAP staining in tumor tissues from the RIL-175 orthotopic model. **f** DHODH protein levels in *DHODH*-knockdown LX-2 cells. **g** Schematic illustration of the tumor model established in BALB/c nude mice by subcutaneous injection of Huh7 cells mixed with *DHODH*-knockdown LX-2 cells at a 1:1 ratio. Created with BioRender.com, agreement number: BL29JVSSBF. **h** Tumor growth curves in nude mice treated under four conditions: vehicle control, sorafenib (5 mg/kg), Huh7 co-injected with sh*Ctrl* LX-2, or Huh7 co-injected with *shDHODH*-LX-2 cells in combination with sorafenib (*n* = 5 per group). **i**, **j** Immunohistochemical staining (**i**) and quantification (**j**) of Ki67, COL1A1, FN1, and 4-HNE in tumor tissues from nude mice (*n* = 5 per group). Scale bar: 75 μm. Data are presented as the mean ± SEM of independent mice. *p < 0.05; **p < 0.01; ***p < 0.001

To assess the impact of tumor-derived lactate and pyrimidine biosynthesis in HSCs on HCC progression and sorafenib responsiveness in vivo, we first evaluated VB-124, a lactate efflux inhibitor, administered either as monotherapy or in combination with sorafenib in an orthotopic RIL-175 tumor model in C57BL/6 mice [39, 40]. VB-124 or sorafenib monotherapy reduced tumor growth, whereas their combination synergistically suppressed tumor growth, without any differences in body weight between groups (Fig. 6a, b; Fig. S9a). This enhanced efficacy was further supported by a marked reduction in intratumoral lactate concentrations following combination therapy (Fig. 6c). Mechanistically, combination therapy produced a more pronounced decrease in Ki67 expression and ECM deposition (COL1A1, FN1, and Picrosirius red staining) while also increasing markers indicative of lipid peroxidation, including elevated 4-hydroxynonenal (4-HNE) and malondialdehyde (MDA) levels, compared to either treatment alone (Fig. 6d; Fig. S9b-f). Combined VB-124 and sorafenib treatment significantly reduced rRNA intensity in GFAP⁺ cells (Fig. 6e; Fig. S9g). Given prior reports linking lactate transport to T cell function [39], we examined whether inhibition of lactate efflux influences T cell–mediated cytotoxicity. Silencing of *MCT4* did not enhance T cell–mediated cell death, suggesting that the therapeutic efficacy of lactate inhibition in this setting is primarily driven by disruption of the mTORC1-CAD-ECM axis rather than direct T cell activation (Fig. S9h, i).

Next, we examined whether inhibition of pyrimidine synthesis in HSCs could potentiate the antitumor efficacy of sorafenib by subcutaneously injecting BALB/c nude mice with a mixture of Huh7 cells and *DHODH*-knockdown LX-2 cells. Tumors derived from Huh7 cells co-injected with *DHODH*-knockdown LX-2 cells exhibited significantly slower growth than those formed with wild-type LX-2 cells (Fig. 6f–h). Similarly, combining sorafenib treatment with *DHODH*-knockdown LX-2 cells resulted in substantial tumor growth inhibition, with no significant changes in body weight across groups (Fig. 6h; Fig. S9j). In tumor tissues derived from Huh7 cells mixed with *DHODH*-knockdown LX-2 cells, Ki67, COL1A1, and FN1 expression was reduced, whereas markers associated with lipid peroxidation were increased; these effects were further amplified upon sorafenib co-treatment (Fig. 6i, j; Fig. S9k, l). Immunofluorescence analysis revealed a marked reduction in rRNA intensity in GFAP + cells in tumors derived from *DHODH*-knockdown LX-2 cell mixtures, and sorafenib co-treatment led to an even more pronounced decrease (Fig. S9m, n). Taken together, these findings indicate that inhibiting lactate-mediated pyrimidine biosynthesis effectively suppresses ECM production, enhances ferroptosis, and improves the antitumor efficacy of sorafenib, suggesting a promising metabolic vulnerability for therapeutic intervention in HCC.

## Discussion

**Fig. 7:**
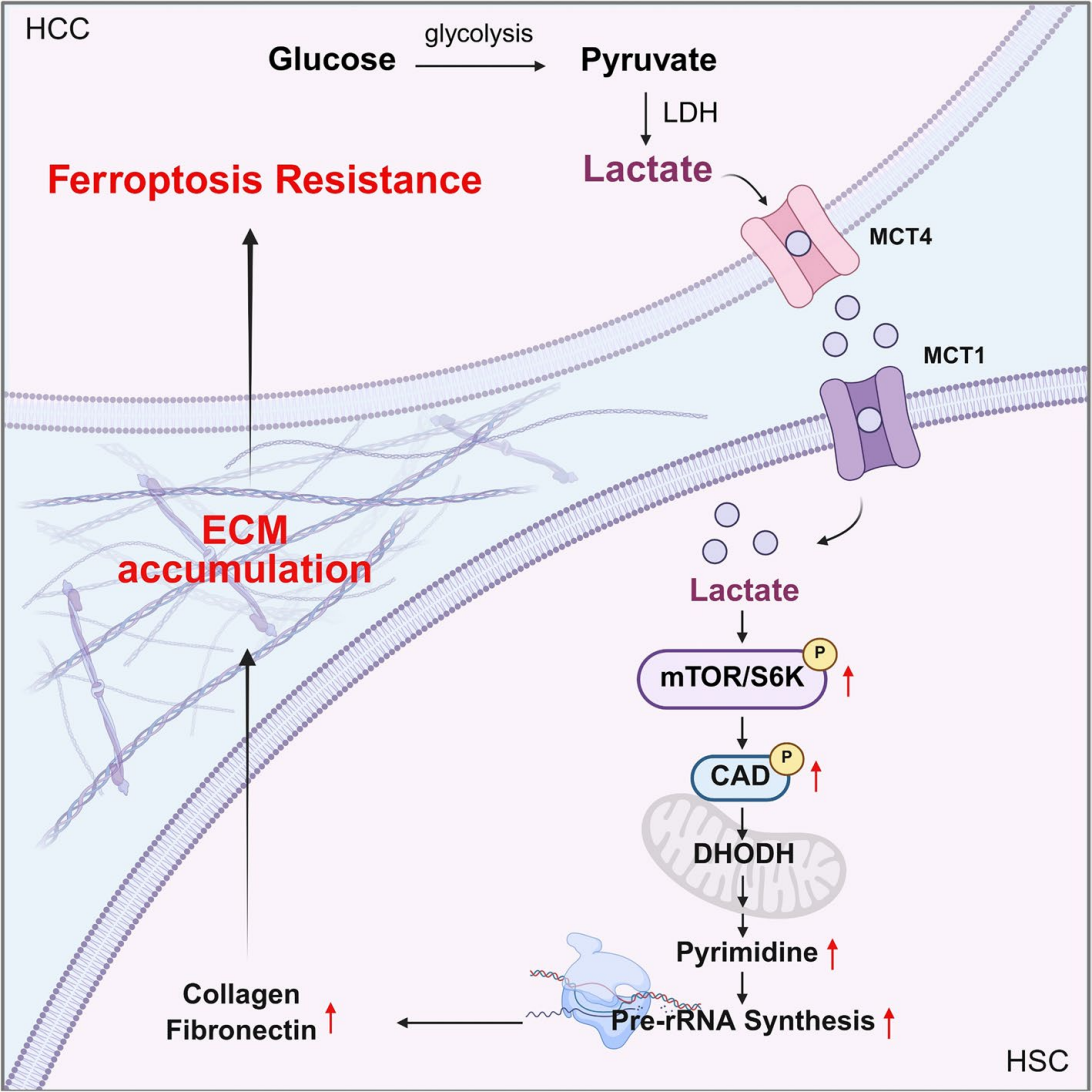
Effects of lactate-driven pyrimidine synthesis on ferroptosis resistance in liver cancer. Schematic illustration of metabolic crosstalk between HCC cells and HSCs within the tumor microenvironment. Lactate released by HCC cells enters HSCs via MCT1, where it activates mTORC1–CAD signaling to induce pyrimidine biosynthesis. Lactate-fueled pyrimidine synthesis drives pre-rRNA generation and supports translation of ECM proteins, leading to ECM accumulation. The deposited ECM protects HCC cells from drug-induced ferroptosis, thereby contributing to therapeutic resistance. Created with BioRender.com, agreement number: EF29JZT3AR

In this study, we elucidated how lactate drives ECM production in HSCs through mTORC1-dependent pyrimidine biosynthesis. This cascade involves sequential activation of CAD and DHODH, which increases rRNA synthesis and ribosomal biogenesis. Inhibiting lactate efflux from cancer cells or suppressing pyrimidine biosynthesis in HSCs effectively disrupted lactate-driven ECM production and restored sensitivity to sorafenib-induced ferroptosis (Fig. 7). These findings suggest that targeting pyrimidine biosynthesis in HSCs may represent a promising strategy to mitigate ECM accumulation and overcome drug resistance in HCC.

Lactate, once regarded merely as a metabolic byproduct of glycolysis, is now recognized as an active signaling molecule within the TME. Studies have shown that lactate modulates immune responses and angiogenesis, although its role in ECM remodeling remains poorly understood [41]. Here, we extend previous findings linking mTORC1-driven de novo pyrimidine synthesis to S6 kinase 1–mediated CAD phosphorylation [42] by showing that cancer-derived lactate increases mTORC1 activation and subsequent CAD phosphorylation in HSCs. This signaling cascade drives upregulation of ECM proteins, including COL1A1 and FN1, thereby fostering a fibrotic, tumor-supportive niche in HCC. A strong positive correlation between lactate-mediated pyrimidine biosynthesis and ECM deposition was consistently observed in tumor tissues from patients with HCC. In particular, upregulation of lactate-related and ECM-associated genes, together with higher p-CAD intensity in HSCs, was strongly associated with poorer survival outcomes. Thus, our findings imply that pyrimidine biosynthesis–driven ECM remodeling as a previously unrecognized mechanism linking tumor-derived lactate to stromal reprogramming and ferroptosis resistance, distinguishing this work from earlier studies focused on immune or angiogenic effects [16].

Nucleotide availability preferentially supports production of rRNA over mRNA in proliferating cells, and pyrimidine biosynthesis plays an essential role in driving rRNA biogenesis and protein translation [33]. CAD promotes pyrimidine synthesis and pre-rRNA production, thereby supporting protein translation in rapidly proliferating cells, as exemplified by memory CD8 + T cells during immune responses. CAD supports ribosomal biogenesis and thus plays a crucial role in cytokine responses upon rechallenge, ensuring sustained immune activation through translational regulation [43]. Similarly, inhibition of DHODH, a rate-limiting enzyme in the de novo pyrimidine synthesis pathway, causes nucleolar stress and impairs rRNA transcription in glioblastoma cells, underscoring the dependence of rapidly proliferating cells on sustained pyrimidine production to maintain ribosomal biogenesis [33]. We extended these findings by showing that HCC cell-derived lactate drives ECM production through a DHODH-dependent mechanism in which uridine, an essential precursor of pre-rRNA synthesis, supports nascent ECM protein synthesis. These results provide mechanistic insight into how cancer cell-derived lactate promotes ECM accumulation via pyrimidine synthesis and pre-rRNA production, thereby identifying a possible driver of HCC progression.

Excessive ECM deposition by activated HSCs plays a crucial role in shaping the HCC TME, not only by promoting tumor growth but also by conferring resistance to ferroptosis. Accumulating evidence suggests that ferroptosis resistance is a common feature of multiple cancer types, including HCC, and that evasion of ferroptosis underlies reduced sensitivity to antitumor therapies [4]. We found that ECM production mediated by pyrimidine biosynthesis confers resistance to sorafenib-induced ferroptosis in HCC cells. Consistent with recent findings implicating collagen-DDR1 signaling in HCC progression via repression of Hippo pathway activity [38], our results indicate that collagen treatment activates YAP via DDR1, rendering HCC cells resistant to sorafenib-induced ferroptosis. This suggests that COL1A1 not only serves as a physical barrier but also actively modulates Hippo-YAP signaling to promote HCC cell survival. We found that blocking lactate efflux from HCC cells or inhibiting DHODH in HSCs results in a marked reduction in ECM production and enhances ferroptosis susceptibility in vivo. These results imply stromal pyrimidine metabolism could serve as a potential therapeutic vulnerability, highlighting the importance of targeting metabolic crosstalk between tumor cells and HSCs.

Despite these promising findings, several limitations should be acknowledged. First, although MCT4 inhibition has been reported to modulate the tumor immune microenvironment [39], the antitumor effects observed in our model appear to arise primarily from reduced ECM production. Such stromal alterations may indirectly shape immune cell accessibility and function within the tumor microenvironment, highlighting the need for more comprehensive investigation into how MCT4 inhibition coordinates ECM regulation and immune modulation. Furthermore, additional stromal or immune components may also contribute to ECM-driven ferroptosis resistance, necessitating further study of these complex interactions. Second, although we identified 4-HNE and MDA as markers of lipid peroxidation associated with ferroptosis, in vivo ferroptosis markers remain limited in specificity. Our findings should therefore be interpreted cautiously as supportive rather than definitive evidence of ferroptosis activation. Third, while our findings support a model in which pyrimidine availability preferentially fuels ribosome biogenesis, the relative contribution of other RNA species could not be directly assessed in the current study. Consequently, more comprehensive dissection of pyrimidine allocation among distinct RNA pools will be required in future investigations. Finally, our patient cohort analysis, while informative, is based on a single-institution dataset and warrants validation in larger, multicenter cohorts to establish the generalizability of p-CAD and lactate-associated signatures as prognostic biomarkers in HCC.

In conclusion, this study identifies a previously unrecognized metabolic axis in which cancer-derived lactate drives ECM production in HSCs through pyrimidine biosynthesis–dependent pre-rRNA generation, fostering a fibrotic, tumor-supportive microenvironment. By proposing a direct mechanistic link between ECM deposition and ferroptosis resistance, our findings provide a compelling rationale for targeting lactate metabolism and pyrimidine biosynthesis—including DHODH inhibition—to disrupt tumor–stroma interactions, overcome drug resistance, and improve therapeutic outcomes in HCC. Future studies should further delineate how this metabolic pathway intersects with immune regulation and evaluate the translational potential of HSC-directed, DHODH-based therapies in clinical settings.

## Materials and methods

### Patients and specimens

Human HCC tissue samples were obtained from 76 patients with HCC who underwent surgical resection at Keimyung University Dongsan Hospital in Daegu, Korea, between 2008 and 2012. This study was approved by the Institutional Review Board (IRB) of Keimyung University Dongsan Hospital (IRB no. 2024–06–033). Lactate concentrations in these samples were measured using the Lactate-Glo™ Assay (Promega, Madison, WI, USA). HCC tissue samples were also collected from a separate cohort of 206 patients who underwent surgical resection at Kyungpook National University Hospital in Daegu, Korea, between 2005 and 2010. This study was approved by the IRB of Kyungpook National University Hospital (IRB no. KNUH-2014–04–056–001). HCC was diagnosed and managed according to the guidelines of the American Association for the Study of Liver Diseases [44]. Patients who underwent preoperative anticancer treatment, including transarterial chemoembolization or local ablation therapy, were excluded from the study. Clinical information—including age, sex, tumor size, tumor number, laboratory findings, and the etiology of underlying liver disease—was collected through review of medical records. Written informed consent was obtained from all participants.

### Cell line culture and preparation of Conditioned medium (CM) from liver cancer cells

The human hepatic stellate cell line LX-2 (Sigma, St. Louis, MO, USA) was cultured in Dulbecco’s Modified Eagle Medium (DMEM) containing 10% fetal bovine serum (FBS) and 1% penicillin/streptomycin (P/S). The human liver cancer cell lines Huh7 (Korean Cell Line Bank, Seoul, Korea) and PLC/PRF/5 (ATCC, Manassas, VA, USA) were cultured in DMEM and Roswell Park Memorial Institute (RPMI) 1640 media containing 10% FBS and 1% P/S. The human monocytic cell line THP-1 (Korean Cell Line Bank) was cultured in RPMI 1640 medium containing 10% FBS and 1% P/S. The mouse liver cancer cell line RIL-175 (a kind gift from Lars Zender at University Hospital Tübingen, Germany) was cultured in RPMI 1640 medium containing 10% FBS and 1% P/S. All cells were incubated at 37 °C in a humidified atmosphere of 5% CO_2_. Short tandem repeat profiling was performed to authenticate all human cell lines, and mycoplasma contamination was assessed using the MycoStrip Mycoplasma Detection Kit (InvivoGen, San Diego, CA, USA), confirming that all cells were free of contamination. To prepare CM from liver cancer cells, 2 × 10^6^ cells were seeded in a 100 mm dish. The following day, the medium was replaced with serum-free medium, and the cells were incubated for 24 h. The CM was then harvested and filtered through a 0.2 μm pore-size filter. Boiled CM was warmed to 100 °C for 15 min and then filtered to remove precipitate.

### Measurement of lactate

Lactate levels were measured in CM and boiled CM derived from Huh7 and PLC/PRF/5 cells and in homogenized human HCC tumor and non-tumor tissues. Lactate content was measured using the Lactate-Glo™ Assay (Promega) following the manufacturer’s instructions. Data were normalized to tissue weight.

### Chemical treatments

LX-2 cells were treated with CM, sodium L-lactate (10 mM; Sigma), AR–C 155858 (2 µM; MedChemExpress, Monmouth Junction, NJ, USA), rapamycin (20 nM; Sigma), PALA (150 µM; MedChemExpress), BRQ (0.1 µM; Selleckchem, Houston, TX, USA), uridine (5, 10, 50 µM; Sigma), cytidine (50 µM, Sigma), mitoQH_2_ (0.1 µM; Cayman, Ann Arbor, MI, USA), and CX-5461 (0.1 µM; MedChemExpress) for 24 h. Huh7 and PLC/PRF/5 cells were treated with IKE (1 µM; Selleckchem) and sorafenib (10 µM; Selleckchem) for 6 h, and ferrostatin-1 (1 µM, Sigma), Z-VAD-FMK (20 µM; Selleckchem), necrostatin-1 (20 µM; MedChemExpress), and MGH-CP1 (2 µM, MedChemExpress) were applied for 24 h. For collagen and fibronectin stimulation, cells were serum-starved for 10 h and then treated with collagen I (30 µg/mL; Corning, NY, USA) or fibronectin (30 µg/mL; R&D Systems, MN, USA) for 24 h in the presence or absence of verteporfin (1 µM; MedChemExpress).

### Small interfering RNA (siRNA) transfection

Cells were transfected for 48 h with control siRNA or siRNAs targeting human genes, including LDHA, MCT4, LDHB, HIF-1α, EP300, RICTOR, RPTOR, S6K, CAD, eIF4E, MCT1, DHODH, and DDR1 (Bioneer, Daejeon, Korea), using Lipofectamine RNAiMAX (Thermo Fisher Scientific, Waltham, MA, USA).

### Western blot analysis

Total protein lysates were resolved on Tris–Glycine gels or NuPAGE 4%–12% gels (Thermo Fisher Scientific) and transferred to PVDF membranes. The membranes were blocked with 5% skim milk in TBST (Tris-buffered saline containing 0.1% Tween 20) for 1 h at room temperature and then incubated overnight at 4 °C with primary antibodies diluted in TBST supplemented with 5% bovine serum albumin (BSA). Antibodies specific for the detection of the following proteins were used: COL1A1, HIF-1α, phospho-p70 S6 kinase (Thr389), p70 S6 kinase, p-4E-BP1 (Ser65), 4E-BP1, p-YAP (Ser127), YAP, SLC7A11, GPX4, GAPDH, Histone H3, p-CAD (Ser1859), CAD, HSP90 (all from Cell Signaling Technology, Danvers, MA, USA), fibronectin (BD Biosciences, San Jose, CA, USA), MCT1, DHODH (Proteintech, Chicago, IL, USA), ACSL4, MCT4 (Santa Cruz Biotechnology, Dallas, TX, USA), H3K18la (Abclonal, Woburn, MA, USA), β-actin and puromycin (Sigma). The secondary antibody was anti-rabbit/mouse IgG (GeneTex, Irvine, CA, USA).

### Co-culture experiments

HCC and LX-2 cells were co-cultured at a 1:1 ratio. For indirect co-culture, HCC cells were seeded in the upper chamber and LX-2 cells were plated in the lower chamber of a 24 mm Transwell plate (Corning) and incubated for 24 h. In three-dimensional experiments, co-cultures were maintained in Ultra-Low Attachment (ULA) 96-well plates (Corning) to form spheroids. These spheroids were treated with or without uridine every 2 days for 7–14 days. Following incubation, the spheroids were fixed with 3.7% PFA (Biosesang, Seongnam-si, Korea), permeabilized (0.5% Triton X-100), and incubated overnight at 4 °C with primary antibodies against COL1A1, FN1, YAP, and xCT. Immunofluorescence was visualized using an Olympus FLUOVIEW FV1000 confocal microscope (Olympus, Shinjuku-ku, Tokyo, Japan). For two-dimensional extracellular matrix analysis, HCC and LX-2 cells were seeded on 12 mm coverslips pre-coated with gelatin and glutaraldehyde. The culture medium, supplemented with 50 μg/mL ascorbic acid, was replenished every 2 days for 5–7 days. To isolate the matrix, cells were removed using an extraction buffer (3% Triton X-100 and 0.1% SDS in Ca^2+^/Mg^2+^-free DPBS). The remaining decellularized matrix was then processed for immunofluorescence or stained with a Picro Sirius Red Stain Kit (Abcam, Cambridge, UK) according to the manufacturer’s instructions.

### Animal experiments

Six-week-old male BALB/c nude and C57BL/6 mice were purchased from JABIO (Seoul, Korea). To evaluate the in vivo effects of *DHODH* knockdown, LX-2 cells were transfected with sh*DHODH*. To establish a tumor model mimicking the interaction between hepatoma cells and HSCs, Huh7 cells (1 × 10^7^) and LX-2 cells (1 × 10^7^) were mixed in a 200 μL suspension and injected into the right flank of BALB/c nude mice. Once tumor volumes reached approximately 100 mm^3^, mice (*n* = 5 per group) received daily intraperitoneal (i.p.) injections of vehicle (5% DMSO, 40% PEG300, 5% Tween80, and 50% ddH_2_O) or sorafenib (5 mg/kg; Selleckchem) for 15 days. For intrahepatic inoculation of mouse liver cancer cells, RIL-175 cells (1 × 10^5^) were injected into the left lobe of the liver of C57BL/6 mice (*n* = 6 per group). After injection, the abdominal wall and skin incision were closed with surgical sutures. Mice were treated daily for 12 days with VB-124 (10 mg/kg; MedChemExpress) dissolved in 0.5% methylcellulose and 0.1% Tween-20 via oral gavage, or with sorafenib (5 mg/kg; Selleckchem) via intraperitoneal injection. The final drug administration was performed 4 h before the experimental endpoint. Body weight was measured every 3 days, and tumor volume was calculated as length × width^2^ × 0.5 (mm^3^).

### Statistical analysis

Statistical analyses were performed using GraphPad Prism 8 software. Comparisons between data sets were made using a Student’s t-test, and results are presented as the mean ± SEM. A *p*-value of < 0.05 was considered statistically significant.

## Supplementary Information

Supplementary Material 1.

## Data Availability

The data supporting the findings of this study are included in the article.
