# Supplementary material for: Lactate-driven pyrimidine synthesis promotes ferroptosis resistance in hepatocellular carcinoma
Source: Mol Biomed. 2026 Apr 17;7:53. doi: 10.1186/s43556-026-00450-3 (PMC13090463; doi:10.1186/s43556-026-00450-3)
Supplement: Supplementary file 1 — Supplementary Material 1. [file 43556_2026_450_MOESM1_ESM.docx]

**Supplementary Information**

**Lactate-Driven Pyrimidine Synthesis Promotes Ferroptosis Resistance in Hepatocellular Carcinoma**

Mun-Ju Park, Sebin Lee, Dong-Ho Kim, Mi Kyung Kim, Byoung Kuk Jang, Ghilsuk Yoon, Mihyang Park, Gui-Hwa Jeong, Jun-Kyu Byun, Yeon-Kyung Choi, Keun-Gyu Park

**Supplementary methods**

**RNA isolation**

Total RNA was extracted using the QIAzol reagent (Qiagen, Germantown, MD, USA). After extraction, RNA concentration and purity were measured with a NanoDrop ND-2000 spectrophotometer (Thermo Fisher Scientific, Waltham, MA, USA).

**Quantitative RT-PCR**

Total RNA was isolated using QIAzol reagent, and complementary DNA (cDNA) was synthesized using the RevertAid First Strand cDNA Synthesis kit (Thermo Fisher Scientific). The synthesized cDNA was amplified on a QuantStudio^TM^ 5 Real-Time PCR System (Applied Biosystems, Foster City, CA, USA). The sequences of the primers are listed in Supplementary Table S2. The relative mRNA expression levels were determined using the 2^-ΔΔCT^ method. Gene expression levels were normalized using the endogenous reference gene, 36B4.

**Public data-based gene expression and survival analysis**

Expression profiles for COL1A1 and FN1 were analyzed using TNMplot (https://tnmplot.com/analysis). Single-cell transcriptomic analysis of LDHA expression was performed using HCCDB (http://lifeome.net/database/hccdb2) to compare its expression across cancer, immune, and stromal cells. GEPIA2 (http://gepia2.cancer-pku.cn) was used to analyze overall survival according to the expression levels of COL1A1, FN1, and their combination with LDHA and SLC16A3.

**Measurement of secreted COL1A1 concentration**

To prepare CM from LX-2 cells, 2.5 × 10^5^ cells were seeded in a 60 mm dish. The following day, LX-2 cells were incubated in the presence or absence of sodium L-lactate (10 mM; Sigma) for 24 h. After lactate treatment, the medium was replaced with serum-free medium, and the cells were incubated for an additional 24 h. The CM was then collected and filtered through a 0.2 µm pore-size filter. Secreted COL1A1 levels in the LX-2 CM were quantified using a human COL1A1 ELISA kit (Abclonal, Woburn, MA, USA) according to the manufacturer’s instructions.

**Immunofluorescence analysis**

Cells cultured on glass coverslips were fixed with 4% PFA for 20 min, permeabilized with 0.1% Triton X-100 for 10 min, and blocked with 1% BSA. For tissue microarrays (TMAs), sections were deparaffinized and subjected to antigen retrieval using an IHC-Tek Epitope Retrieval Steamer Set (IHC World, Woodstock, MD, USA). All slides were then incubated with primary antibodies overnight at 4°C, followed by incubation with Alexa Fluor 488-conjugated goat anti-mouse or Alexa Fluor 568-conjugated goat anti-rabbit IgG for 1 h at room temperature. Nuclei were counterstained with DAPI, and immunofluorescence images were captured using an EVOS M5000 Imaging System (Thermo Fisher Scientific) and quantified with ImageJ software.

**Macrophage polarization and collection of Tumor associated macrophages (TAM)-conditioned medium**

THP-1 cells were differentiated into macrophages by treatment with 50 nM phorbol 12-myristate 13-acetate (PMA; Sigma) for 24 h, followed by a 24 h resting period in fresh RPMI 1640 medium. To generate TAMs, PMA-primed THP-1 cells were incubated for 24 h in a 1:1 (v/v) mixture of CM from liver cancer cells and RPMI 1640 containing 10% FBS and 1% penicillin–streptomycin. The medium was then replaced with serum-free RPMI 1640, and the cells were cultured for an additional 24 h. The resulting CM was collected and passed through a 0.2 µm pore-size filter to remove cell debris.

**Measurement of active TGF-β1 concentration**

Active TGF-β1 levels in CM derived from TAMs and liver cancer cells were measured using an Active TGF-β1 ELISA kit (BioLegend, San Diego, CA, USA) according to the manufacturer’s instructions.

**Isolation and culture of primary HSCs**

Primary hepatic stellate cells (pHSCs) were isolated from C57BL/6 mice via in situ liver perfusion through the inferior vena cava at a constant flow rate of 5 mL/min. The liver was sequentially perfused with EGTA-buffer (136.89 mmol/L NaCl, 5.37 mmol/L KCl, 0.64 mmol/L NaH₂PO₄·H₂O, 0.85 mmol/L Na₂HPO₄, 9.99 mmol/L HEPES, 4.17 mmol/L NaHCO₃, 0.5 mmol/L EGTA, and 5 mmol/L glucose, pH 7.35–7.4) for 2 min, followed by enzyme buffer (136.89 mmol/L NaCl, 5.37 mmol/L KCl, 0.64 mmol/L NaH₂PO₄·H₂O, 0.85 mmol/L Na₂HPO₄, 9.99 mmol/L HEPES, 4.17 mmol/L NaHCO₃, and 3.81 mmol/L CaCl₂·2H₂O, pH 7.35–7.4) containing 0.4 mg/mL pronase (Roche Diagnostics, Indianapolis, IN, USA) for 5 min and 0.193 U/mg collagenase (Roche Diagnostics) for 7 min. The excised liver was then digested at 40°C for 20 min with gentle agitation, filtered through a 70 µm nylon mesh, and centrifuged at 580 × g for 10 min. To isolate the HSC fraction, the resulting pellet was resuspended in Gey’s Balanced Salt Solution (GBSS; Sigma) and subjected to density gradient centrifugation using Cell-OptiPrep (Sigma) at 1380 × g for 17 min without braking. HSCs were then harvested from the white interphase, washed with HBSS, and cultured in DMEM supplemented with 10% FBS and 1% P/S.

**Isolation of primary hepatocytes**

Primary hepatocytes were isolated from C57BL/6J mice by in situ liver perfusion through the portal vein at a flow rate of 5 mL/min. The liver was sequentially perfused for 10 min with an EGTA-containing resuspension buffer (5.4 mmol/L KCl, 0.44 mmol/L KH₂PO₄, 140 mmol/L NaCl, 0.34 mmol/L Na₂HPO₄, 0.5 mmol/L EGTA, and 25 mmol/L Tricine, pH 7.2), followed by 10 min perfusion with a collagenase solution (Ca²⁺- and Mg²⁺-free Hank’s Balanced Salt Solution, pH 7.2, containing 0.75 mg/mL collagenase type I; Worthington Biochemical Corp., Freehold, NJ, USA). After perfusion, the excised liver was gently agitated at 37°C for 20 min. The resulting cell suspension was filtered through a 70 µm nylon mesh and centrifuged at 42 × g for 5 min at 4°C. The hepatocyte pellet was then resuspended in William’s Medium E (Sigma) and seeded onto Type I collagen-coated dishes (Corning). After an initial 2–3 h incubation for cell attachment, the medium was replaced with Medium 199 (Sigma).

**Measurement of OCR**

OCR was measured in 24-well plates using the XF-24 Extracellular Flux Analyzer (Seahorse Bioscience, North Billerica, MA, USA). On the following day, cells were treated as indicated, and the sensor cartridge was calibrated. LX-2 cells were washed twice with XF Medium (Seahorse Bioscience) and incubated for 1 h at 37°C in a non-CO_2_ incubator. During OCR measurement, oligomycin (1 μM; Sigma), CCCP (carbonyl cyanide 3-chlorophenylhydrazone, 5 μM; Sigma), antimycin (5 μM; Sigma), or rotenone (1 μM; Sigma) was added at the indicated time points. Data were normalized to the protein concentration in each sample.

**Puromycin incorporation assay**

To assess protein synthesis, cells were treated with 1 µg/mL puromycin (Sigma) for 30 min at 37°C. Protein incorporation was detected using an anti-puromycin antibody (Sigma) by Western blotting and immunofluorescence analysis.

**Nascent protein labeling and pull-down assay**

LX-2 cells were incubated for 24 h in sodium L-lactate (10 mM; Sigma) or HCC-derived CM in the presence or absence of BRQ (0.1 μM; Selleckchem). The medium was then replaced with methionine-free medium (Sigma) containing L-cystine (Sigma), L-glutamine (Sigma), 10% dialyzed FBS (Gibco), and 1% P/S to deplete intracellular methionine. After 30 min, cells were treated with L-azidohomoalanine (AHA; Click Chemistry Tools, Newark, CA, USA) for 4 h to label newly synthesized proteins. Cells were then lysed using RIPA buffer (Thermo Fisher Scientific) with added PMSF (VWR International, Radnor, PA, USA), aprotinin (Sigma), leupeptin (Sigma), and phosphatase inhibitor (Sigma). Lysates were incubated on ice for 20 min. The lysates were then centrifuged at 17,000 × g for 20 min at 4°C, and the supernatants were collected. Nascent proteins were labeled using the Click-&-Go Protein Reaction Buffer Kit (Click Chemistry Tools) with Biotin-alkyne (Click Chemistry Tools). Biotinylated proteins were incubated for 1 h at 4°C with 50 μL of streptavidin-agarose beads (Thermo Fisher Scientific). The beads were washed four times with 1 mL PBS/0.1% SDS, centrifuged at 2500 × g for 2 min, and the supernatant was discarded. The purified proteins were analyzed by Western blotting.

**Measurement of UTP concentrations**

LX-2 cells were incubated with sodium L-lactate (10 mM; Sigma) or HCC-derived CM for 24 h in the presence or absence of BRQ (0.1 μM). Under these conditions, UTP levels were measured using the UTP ELISA kit (MYBioSource, San Diego, CA, USA) according to the manufacturer’s protocol.

**Cell viability assay**

For the cell viability assay, cells were seeded at a density of 1 × 10^4^ cells per well in 96-well plates. Cell viability was assessed using the Cell Counting Kit-8 (CCK-8; Dojindo, Kumamoto, Japan) according to the manufacturer’s instructions. After adding the CCK-8 reagent, the plates were incubated at 37 °C for 1 h, and absorbance was measured at 450 nm using a microplate reader. Cell viability was calculated relative to the untreated control group.

**EthD-1 staining**

For EthD-1 staining, cells were incubated with EthD-1 (4 μM; Biotium, Fremont, CA, USA) for 30 min, followed by three washes with PBS containing Tween 20. Cells were fixed with 4% PFA (Biosesang) for 15 min, followed by three washes with PBS containing Tween 20. Nuclei were then stained with DAPI. Data are presented as the area of red fluorescence in cells.

**Detection of lipid ROS**

For fluorescence detection of lipid ROS, cells were incubated with C11-BODIPY (10 μM; Thermo Fisher Scientific) for 30 min, followed by three washes with PBS. Nuclei were subsequently stained with NucBlue™ Live ReadyProbes™ Reagent (Thermo Fisher Scientific). Data are expressed as the ratio of green to red fluorescence intensity within the cells.

**Cytoplasmic/Nuclear Fractionation Assay**

For subcellular fractionation, cytoplasmic and nuclear extracts were prepared using the Nuclear Extraction Kit (Abcam) according to the manufacturer’s instructions. Briefly, cells were collected using a cell scraper and pelleted by centrifugation. The pellets were resuspended in the provided pre-extraction buffer and centrifuged, and the supernatant containing the cytoplasmic fraction was collected. The remaining pellet was washed, centrifuged again, and resuspended in nuclear extraction buffer to obtain the nuclear fraction. Protein concentrations in each fraction were determined using the Pierce BCA Protein Assay Kit (Thermo Fisher Scientific). Equal amounts of protein from the cytoplasmic and nuclear fractions were resolved by SDS–PAGE and subjected to immunoblot analysis. The purity of each fraction was validated by immunoblotting for GAPDH (cytoplasmic marker) and Histone H3 (nuclear marker).

**Dual luciferase assay**

Cells were seeded in a 24-well plate at a density of 5 × 10^4^ cells per well. The following day, cells were co-transfected with 475 ng of the 8xGTIIC firefly luciferase reporter plasmid (Addgene, Watertown, MA, USA) and 25 ng of the pRL-SV40P Renilla luciferase control plasmid (Addgene) using the TransIT-LT1 transfection reagent (Mirus Bio LLC, Madison, WI, USA). After transfection, cells were treated with collagen I (Corning). Luciferase activities were measured using the Dual-Luciferase Reporter Assay System (Promega, Madison, WI, USA) according to the manufacturer’s instructions. Firefly luciferase activity was normalized to Renilla luciferase activity.

**Generation of stable cell lines**

To generate stable *DHODH*-knockdown LX-2 cells, a control short hairpin RNA (shRNA) and sh*DHODH* (shRNA targeting *DHODH*) lentiviral vector were purchased from VectorBuilder (Chicago, IL, USA). Lentiviral particles were produced by transfecting HEK293T cells with the vectors using Lenti-X Packaging Single Shots (Takara, San Jose, CA, USA). After 48 h, the lentiviral supernatant was harvested, and viral titers were determined using the Lenti-X p24 Rapid Titer Kit (Takara). LX-2 cells were then infected with concentrated viral supernatant and 8 μg/mL polybrene (Sigma) for 24 h. Transduced cells were selected with 1.5 μg/mL puromycin (Sigma) for a week to generate stable cell lines.

**Immunohistochemistry**

Tumor tissues were harvested at experimental endpoints, fixed in 4% PFA (Biosesang), and paraffin-embedded. Sections were deparaffinized and subjected to antigen retrieval using an IHC-Tek Epitope Retrieval Steamer Set (IHC World, Woodstock, MD, USA). Slides were then incubated overnight at 4 °C with primary antibodies against COL1A1 (Cell Signaling Technology), Ki67 (Thermo Fisher Scientific), fibronectin (BD Biosciences), 4-HNE (R&D Systems), and MDA (Abcam). Signal detection was performed using the SignalStain® Boost IHC Detection Reagent (Cell Signaling Technology) and the Dako Liquid DAB+ Substrate Chromogen System (Agilent Technologies, Santa Clara, CA, USA). Nuclei were counterstained with Dako hematoxylin (Agilent Technologies). Collagen deposition was evaluated with a Picro Sirius Red Stain Kit (Abcam) according to the manufacturer’s instructions. For immunofluorescence, slides were incubated overnight at 4°C with primary antibodies, followed by incubation with Alexa Fluor 568-conjugated goat anti-mouse or Alexa Fluor 488-conjugated goat anti-rabbit IgG for 1 h at room temperature. Nuclei were then counterstained with DAPI, and all stained sections were visualized and quantified using ImageJ software.

**Isolation of mouse CD8^+^ T cells**

Mouse CD8^+^ T cells were isolated from the spleens of 7–8-week-old male C57BL/6 mice using an EasySep^TM^ Mouse CD8^+^ T Cell Isolation Kit (STEMCELL Technologies, Vancouver, BC, Canada) according to the manufacturer’s instructions.

**T cell–mediated tumor cell killing assay**

Mouse CD8⁺ T cells were activated with anti-CD3 (5 μg/mL; BioLegend, San Diego, CA, USA) and anti-CD28 (2 μg/mL; BioLegend) antibodies in the presence of IL-2 (10 ng/mL; BioLegend). Activated CD8⁺ T cells were then co-cultured with RIL-175 cells at a 1:1 ratio for 24 h.

**Assessment of cell death**

To assess cell death in RIL-175 cells, cells were incubated with 1 μg/mL propidium iodide (PI; Sigma) in PBS for 5 min and analyzed on a BD Accuri C6 flow cytometer (BD Biosciences, San Jose, CA, USA).


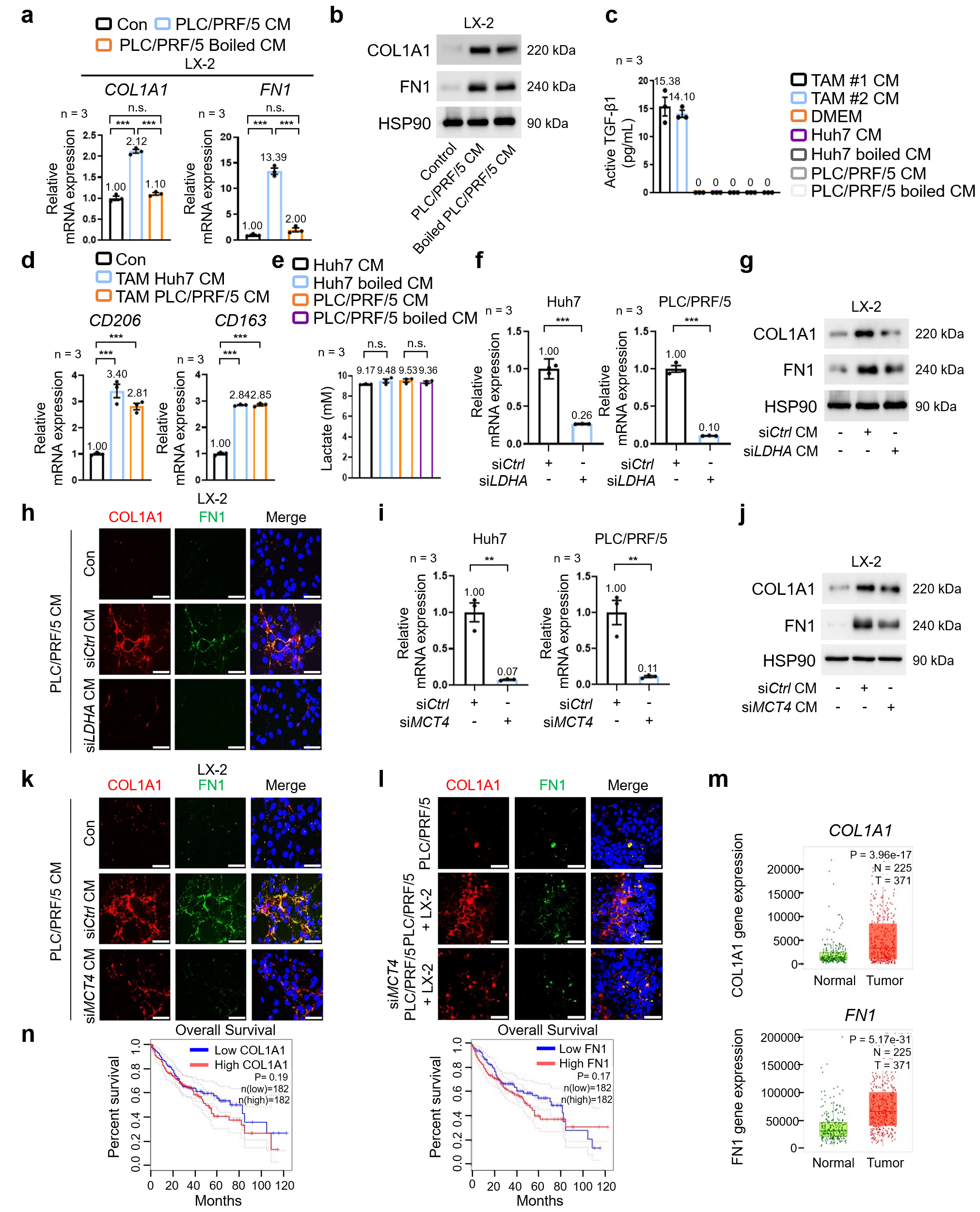
**Supplementary Figure 1 (Related to Fig. 1). Effects of *LDHA* or *MCT4* silencing on ECM production in HSCs.** (a, b) *COL1A1* and *FN1* mRNA expression (a, n = 3) and protein levels (b) in LX-2 cells cultured for 24 h in CM or boiled CM from PLC/PRF/5 cells. (c) Levels of active TGF-β1 measured in CM or boiled CM from Huh7, PLC/PRF/5 cells, and TAMs (TAM #1, THP-1-derived macrophages treated with Huh7 CM; TAM #2, THP-1-derived macrophages treated with PLC/PRF/5 CM) (n = 3). (d) Relative mRNA expression of *CD206* and *CD163* in TAMs compared with THP-1-derived macrophages (control) (n = 3). (e) Lactate concentrations in CM or boiled CM from Huh7 and PLC/PRF/5 cells (n = 3). (f, i) Validation of *LDHA* (f) and *MCT4* (i) knockdown efficiency in Huh7 (left) and PLC/PRF/5 (right) cells (n = 3). (g, j) COL1A1 and FN1 protein levels in LX-2 cells cultured for 24 h in CM from PLC/PRF/5 cells with *LDHA* knockdown (g) or *MCT4* knockdown (j). (h, k) Representative immunofluorescence images of COL1A1 (red) and FN1 (green) expression in LX-2 cells cultured in *LDHA*-silenced (h) or *MCT4*-silenced (k) CM from PLC/PRF/5 cells. (l) Representative immunofluorescence images of COL1A1 (red) and FN1 (green) in spheroids composed of PLC/PRF/5 cells alone, PLC/PRF/5 cells co-cultured with LX-2 cells, and *MCT4*-silenced PLC/PRF/5 cells co-cultured with LX-2 cells. (m) COL1A1 (top) and FN1 (bottom) expression in HCC tissues compared to normal tissues (tumor: n = 371, normal: n = 225), analyzed using the TNMplot database (https://tnmplot.com/analysis). (n) Kaplan-Meier survival analysis of HCC patients stratified by expression levels of COL1A1 (left, n =182/182) and FN1 (right, n = 182/182), analyzed using the GEPIA2 database (http://gepia2.cancer-pku.cn). P = p-value. Fibronectin 1 is referred to as FN1. Scale bar: 75 µm. Data are presented as the mean ± SEM from at least three independent experiments. n.s., not significant; ***p* < 0.01; ****p* < 0.001.


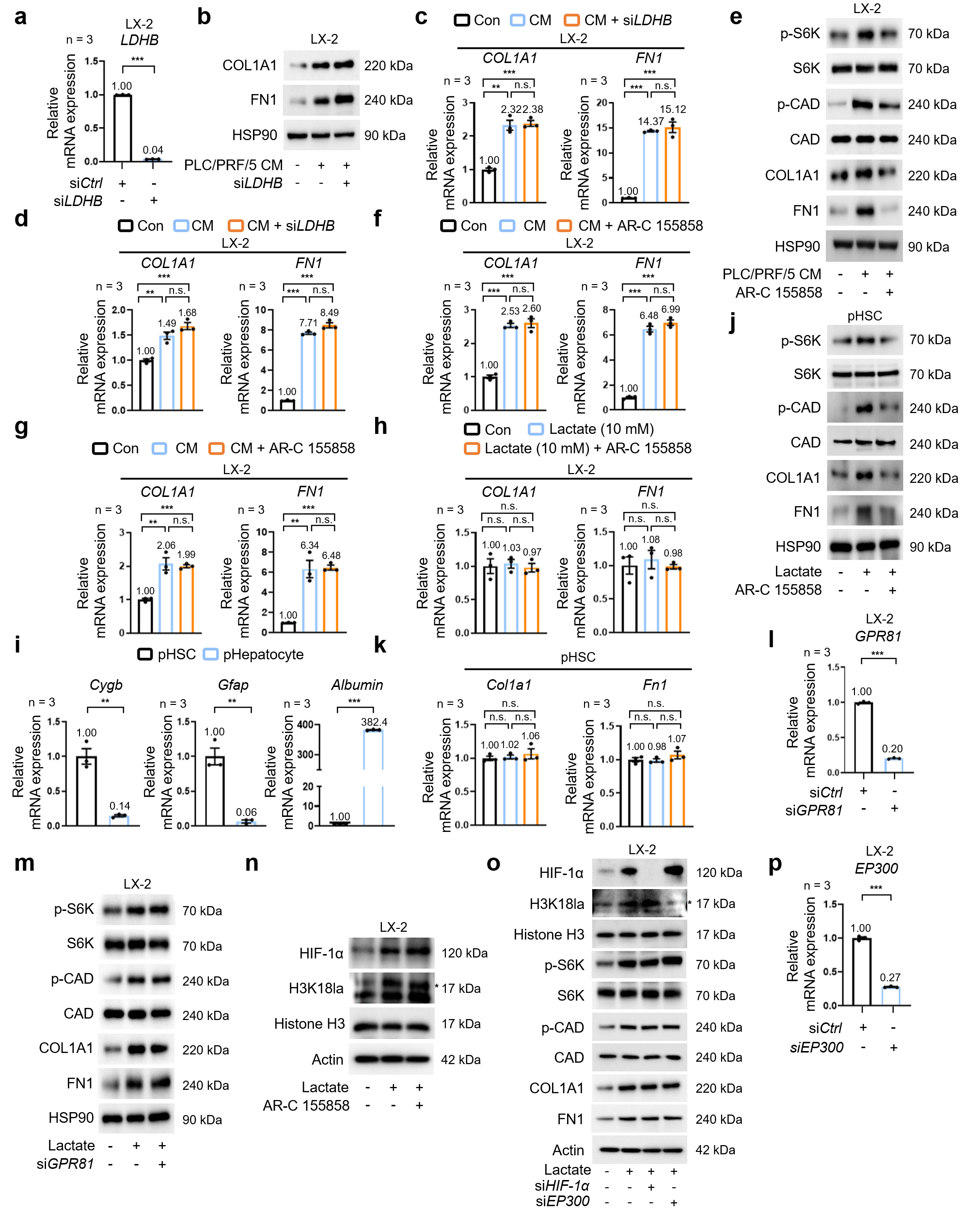
**Supplementary Figure 2 (Related to Fig. 2). Effects of MCT1 inhibition and *GPR81* knockdown on lactate-driven ECM production in HSCs.** (a) Validation of *LDHB* knockdown efficiency in LX-2 cells (n = 3). (b) COL1A1 and FN1 protein levels in *LDHB*-silenced LX-2 cells cultured for 24 h in CM from PLC/PRF/5 cells. (c, d) Relative mRNA expression of *COL1A1* and *FN1* in *LDHB*-silenced LX-2 cells cultured in CM from Huh7 (c) or PLC/PRF/5 cells (d) (n = 3). (e) Effects of AR-C155858 (2 µM, 24 h) on phosphorylated S6K, CAD, COL1A1, and FN1 levels in LX-2 cells cultured in CM from PLC/PRF/5 cells. (f–h) Effects of AR-C155858 (2 µM, 24 h) on *COL1A1* and *FN1* mRNA expression in LX-2 cells cultured in CM or Huh7 (f) and PLC/PRF/5 cells (g), or in the presence or absence of lactate (10 mM, 24 h) (h) (n = 3). (i) Relative mRNA expression of *Cygb, Gfap* and *Albumin* in primary hepatic stellate cells (pHSCs) and primary hepatocytes (pHepatocytes) (n = 3). (j) Effects of AR-C155858 (2 µM, 24 h) on phosphorylated S6K, CAD, COL1A1, and FN1 levels in pHSCs cultured in the presence or absence of lactate (10 mM, 24 h). (k) Effects of AR-C155858 (2 µM, 24 h) on *Col1a1* and *Fn1* mRNA expression in pHSCs cultured in the presence or absence of lactate (10 mM, 24 h) (n = 3). (l) Validation of *GPR81* knockdown efficiency in LX-2 cells (n = 3). (m) Levels of phosphorylated S6K, CAD, COL1A1, and FN1 in *GPR81*-silenced LX-2 cells cultured in the presence or absence of lactate (10 mM, 24 h). (n) Effects of AR-C155858 (2 µM, 24 h) on HIF-1α, H3K18la, and Histone H3 levels in LX-2 cells cultured in the presence or absence of lactate (10 mM, 24 h). (o) HIF-1α, H3K18la, Histone H3, phosphorylated S6K, CAD, COL1A1, and FN1 levels in *HIF-1α*- and *EP300*-silenced LX-2 cells cultured in the presence or absence of lactate (10 mM, 24 h). (p) Validation of *EP300* knockdown efficiency in LX-2 cells (n = 3). Data are presented as the mean ± SEM from at least three independent experiments. n.s., not significant; ***p* < 0.01; ****p* < 0.001. H3K18la, histone H3 lysine 18 lactylation. Asterisk denotes the band corresponding to H3K18la.


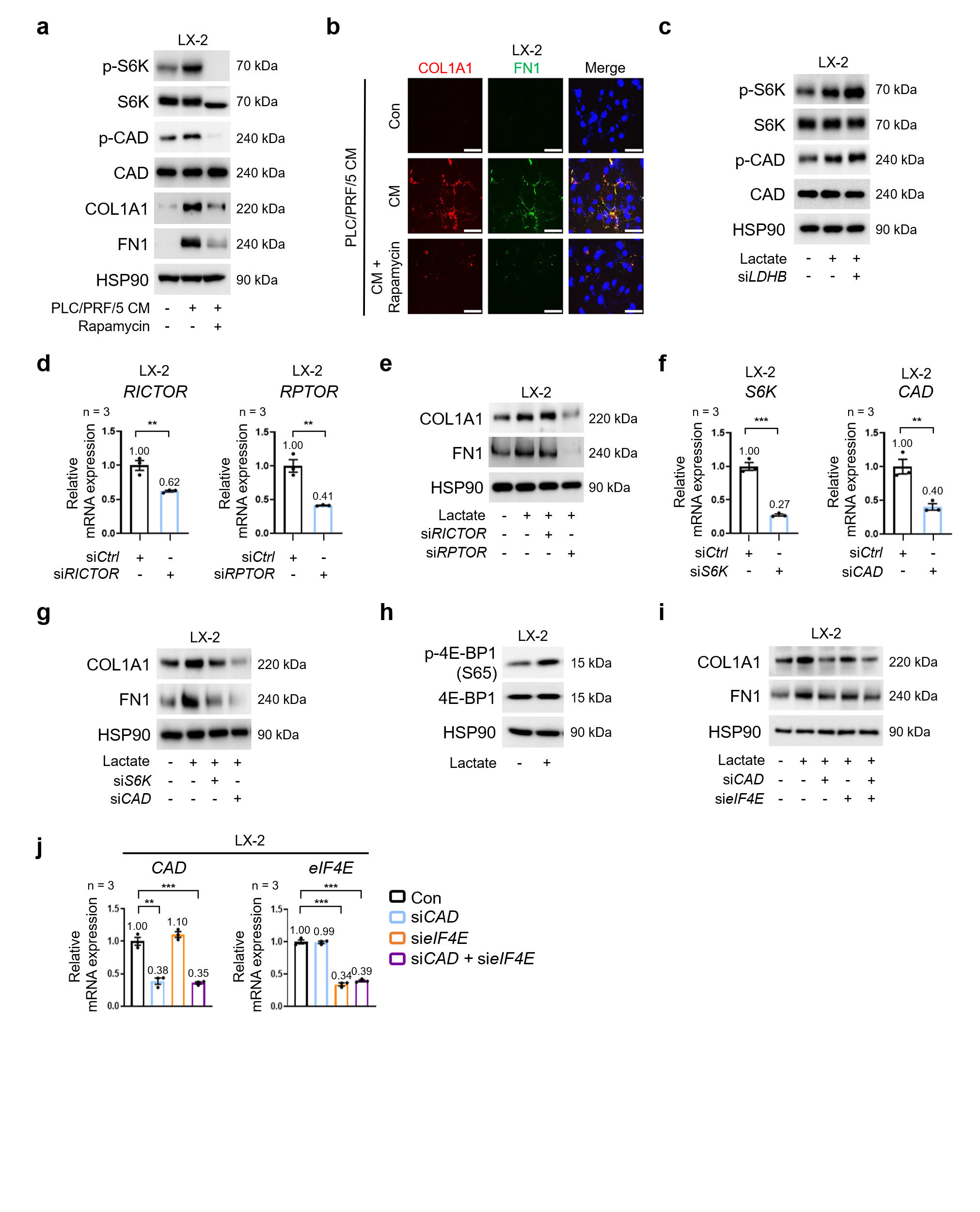
**Supplementary Figure 3 (Related to Fig. 2). Effects of mTORC1 inhibition on lactate-driven ECM production in HSCs.** (a) Effects of rapamycin (20 nM, 24 h) on phosphorylated S6K, CAD, COL1A1, and FN1 levels in LX-2 cells cultured in CM from PLC/PRF/5 cells. (b) Representative immunofluorescence images of COL1A1 (red) and FN1 (green) in LX-2 cells showing the effects of rapamycin (20 nM, 24 h) in CM from PLC/PRF/5 cells. (c) Levels of phosphorylated S6K and CAD in *LDHB*-silenced LX-2 cells cultured in the presence or absence of lactate (10 mM, 24 h). (d) Validation of *RICTOR* (left) and *RPTOR* (right) knockdown efficiency in LX-2 cells (n = 3). (e) COL1A1 and FN1 protein levels in *RICTOR*- and *RPTOR*-silenced LX-2 cells cultured in the presence or absence of lactate (10 mM, 24 h). (f) Validation of *S6K* (left) and *CAD* (right) knockdown efficiency in LX-2 cells (n = 3). (g) COL1A1 and FN1 protein levels in *S6K*- and *CAD*-silenced LX-2 cells cultured in the presence or absence of lactate (10 mM, 24 h). (h) Phosphorylated 4E-BP1(S65) levels in LX-2 cells cultured in the presence or absence of lactate (10 mM, 24 h). (i) COL1A1 and FN1 protein levels in *CAD*- and *eIF4E*-silenced LX-2 cells cultured in the presence or absence of lactate (10 mM, 24 h). (j) Relative mRNA expression of *CAD* and *eIF4E* in *CAD*- and *eIF4E*-silenced LX-2 cells (n = 3). Scale bar: 75 µm. Data are presented as the mean ± SEM from at least three independent experiments. ***p* < 0.01; ****p* < 0.001.


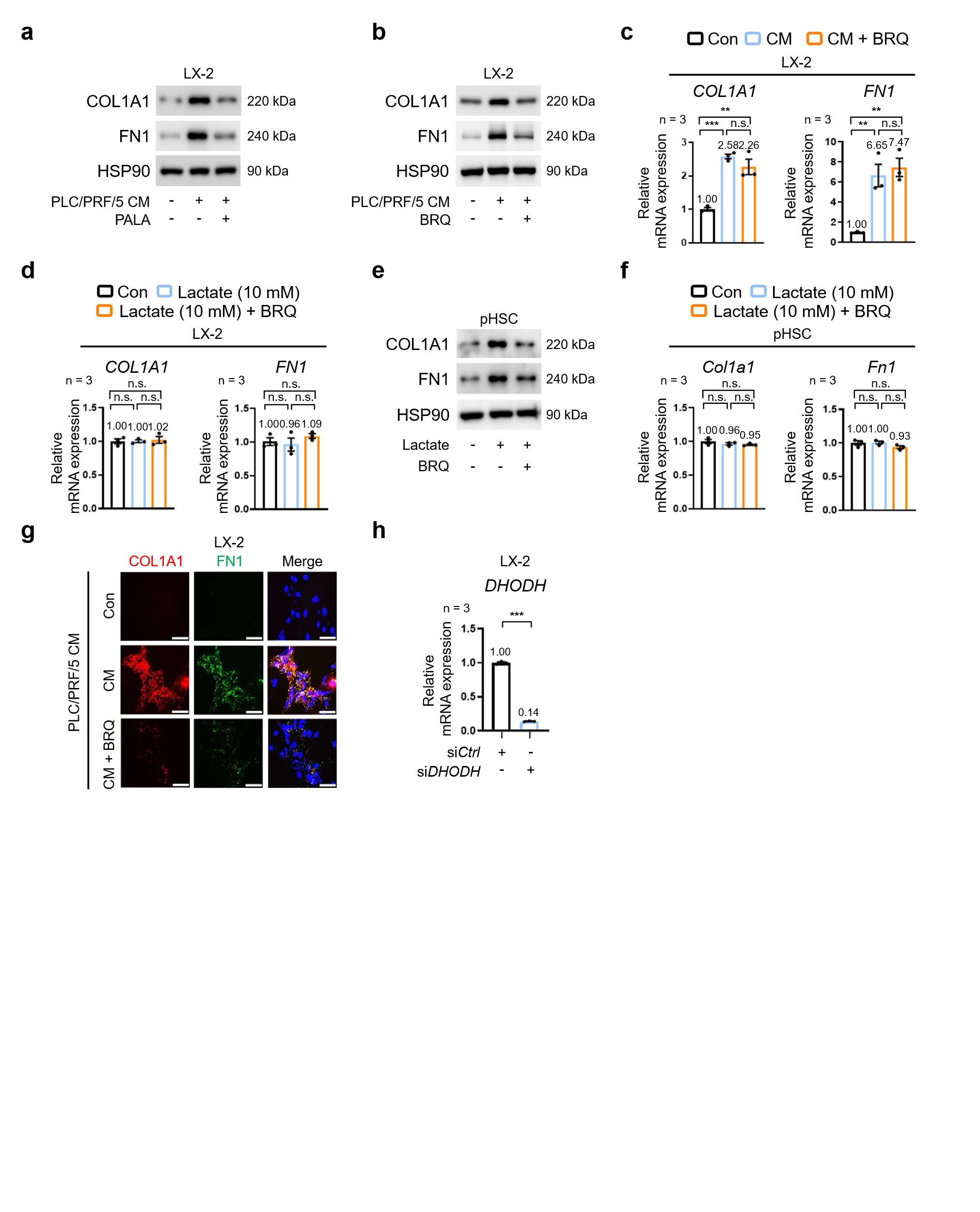
**Supplementary Figure 4 (Related to Fig. 3). Effects of pyrimidine biosynthesis inhibition on ECM production in HSCs induced by HCC-derived CM.** (a) Effects of PALA (150 µM, 24 h) on COL1A1 and FN1 protein levels in LX-2 cells cultured in CM from PLC/PRF/5 cells. (b) Effects of BRQ (0.1 µM, 24 h) on COL1A1 and FN1 protein levels in LX-2 cells cultured in CM from PLC/PRF/5 cells. (c, d) Effects of BRQ (0.1 µM, 24 h) on *COL1A1* and *FN1* mRNA expression in LX-2 cells cultured in CM from PLC/PRF/5 cells (c) or in the presence or absence of lactate (10 mM, 24 h) (d) (n = 3). (e) Effects of BRQ (0.1 µM, 24 h) on COL1A1 and FN1 protein levels in primary hepatic stellate cells (pHSCs) cultured in the presence or absence of lactate (10 mM, 24 h). (f) Effects of BRQ (0.1 µM, 24 h) on *Col1a1* and *Fn1* mRNA expression in pHSCs cultured in the presence or absence of lactate (10 mM, 24 h) (n = 3). (g) Representative immunofluorescence images of COL1A1 (red) and FN1 (green) in LX-2 cells showing the effects of BRQ (0.1 µM, 24 h) in CM from PLC/PRF/5 cells. (h) Validation of *DHODH* knockdown efficiency in LX-2 cells (n = 3). Scale bar: 75 µm. Data are presented as the mean ± SEM from at least three independent experiments. n.s., not significant; ***p* < 0.01; ****p* < 0.001. PALA, N-phosphonacetyl-L-aspartate; BRQ, brequinar.


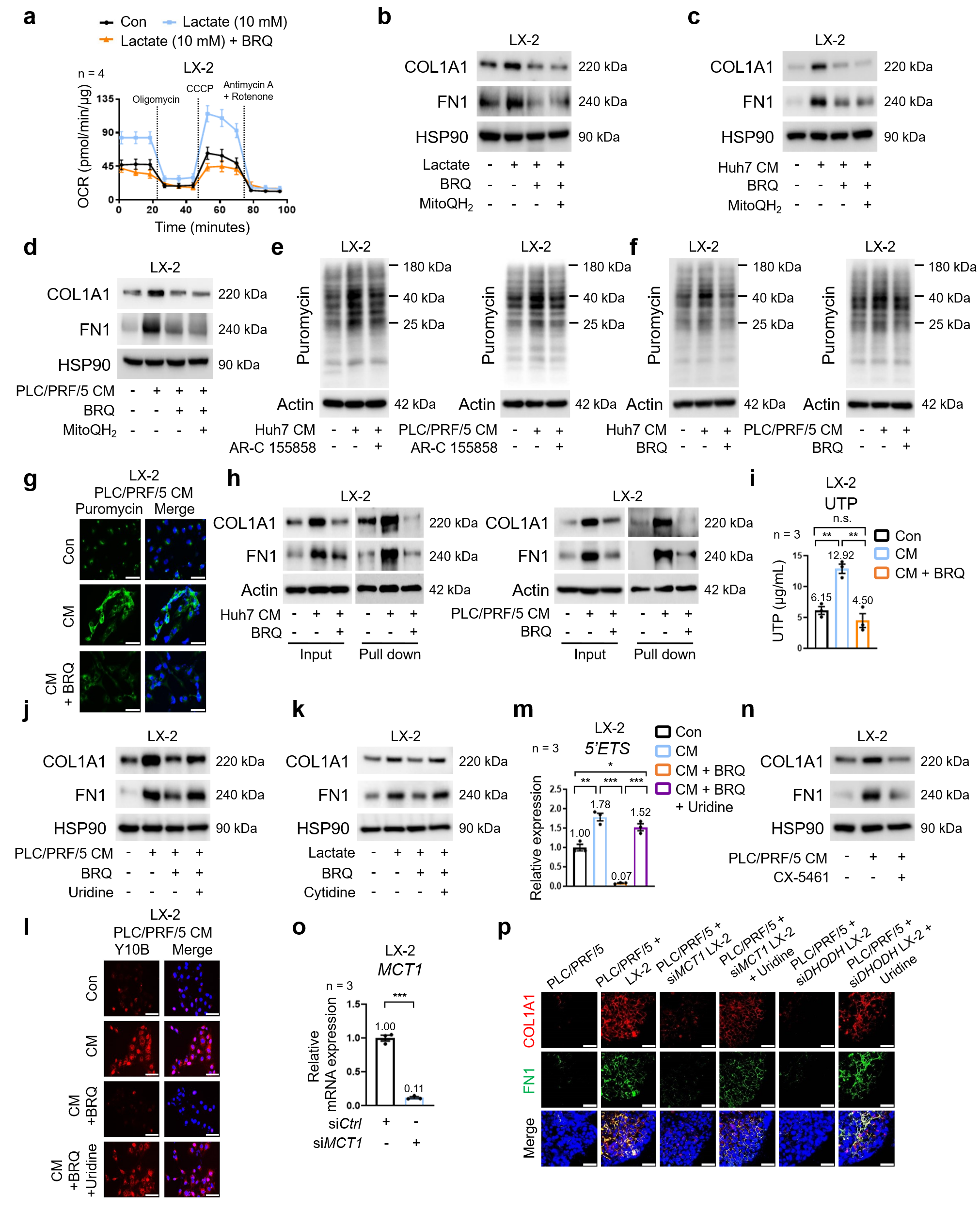
**Supplementary Figure 5 (Related to Figure 4). Effects of mitochondrial distress and pre-rRNA synthesis on lactate-driven ECM production.** (a) Effects of lactate (10 mM, 24 h) and BRQ (0.1 µM) on OCR in LX-2 cells (n = 4). (b–d) Effects of BRQ (0.1 µM, 24 h) or MitoQH_2_ (0.1 µM, 24 h) on COL1A1 and FN1 protein levels in LX-2 cells treated with lactate (10 mM, 24 h) (b) or cultured in CM from Huh7 (c) and PLC/PRF/5 (d) cells. (e, f) Effects of AR-C155858 (2 µM, e) or BRQ (0.1 µM, f) exposure for 24 h on puromycin incorporation in LX-2 cells cultured in CM from Huh7 (left) or PLC/PRF/5 (right) cells. (g) Representative immunofluorescence images of puromycin (1 µg/mL) in LX-2 cells showing the effects of BRQ (0.1 µM, 24 h) in CM from PLC/PRF/5 cells. (h) Pull-down assay showing the effects of BRQ (0.1 µM, 24 h) on COL1A1 and FN1 protein levels in LX-2 cells cultured in CM from Huh7 (left) and PLC/PRF/5 (right) cells. (i) UTP levels in LX-2 cells treated with BRQ (0.1 µM, 24 h) in CM from PLC/PRF/5 cells (n = 3). (j) Effects of BRQ (0.1 µM, 24 h) or uridine (50 µM, 24 h) on COL1A1 and FN1 protein levels in LX-2 cells cultured in CM from PLC/PRF/5 cells. (k) Effects of BRQ (0.1 µM, 24 h) or cytidine (50 µM, 24 h) on COL1A1 and FN1 protein levels in LX-2 cells cultured in the presence or absence of lactate (10 mM, 24 h). (l) Representative immunofluorescence images of Y10B staining in LX-2 cells showing the effects of BRQ (0.1 µM, 24 h) or uridine (50 µM, 24 h) in CM from PLC/PRF/5 cells. (m) Relative expression of *5′ETS* in LX-2 cells treated with BRQ (0.1 µM, 24 h) or uridine (50 µM, 24 h) in CM from PLC/PRF/5 cells (n = 3). (n) Effects of CX-5461 (0.1 µM, 24 h) on COL1A1 and FN1 protein levels in LX-2 cells cultured in CM from PLC/PRF/5 cells. (o) Validation of *MCT1* knockdown efficiency in LX-2 cells (n = 3). (p) Effects of *MCT1*- or *DHODH*-targeting siRNA on COL1A1 (red) and FN1 (green) immunofluorescence staining in LX-2 cells co-cultured with PLC/PRF/5 cells and treated with uridine (50 µM, 24 h). Scale bar: 75 µm. Data are presented as the mean ± SEM from at least three independent experiments. n.s., not significant; **p* < 0.05; ***p* < 0.01; ****p* < 0.001. OCR, oxygen consumption rate; BRQ, brequinar.


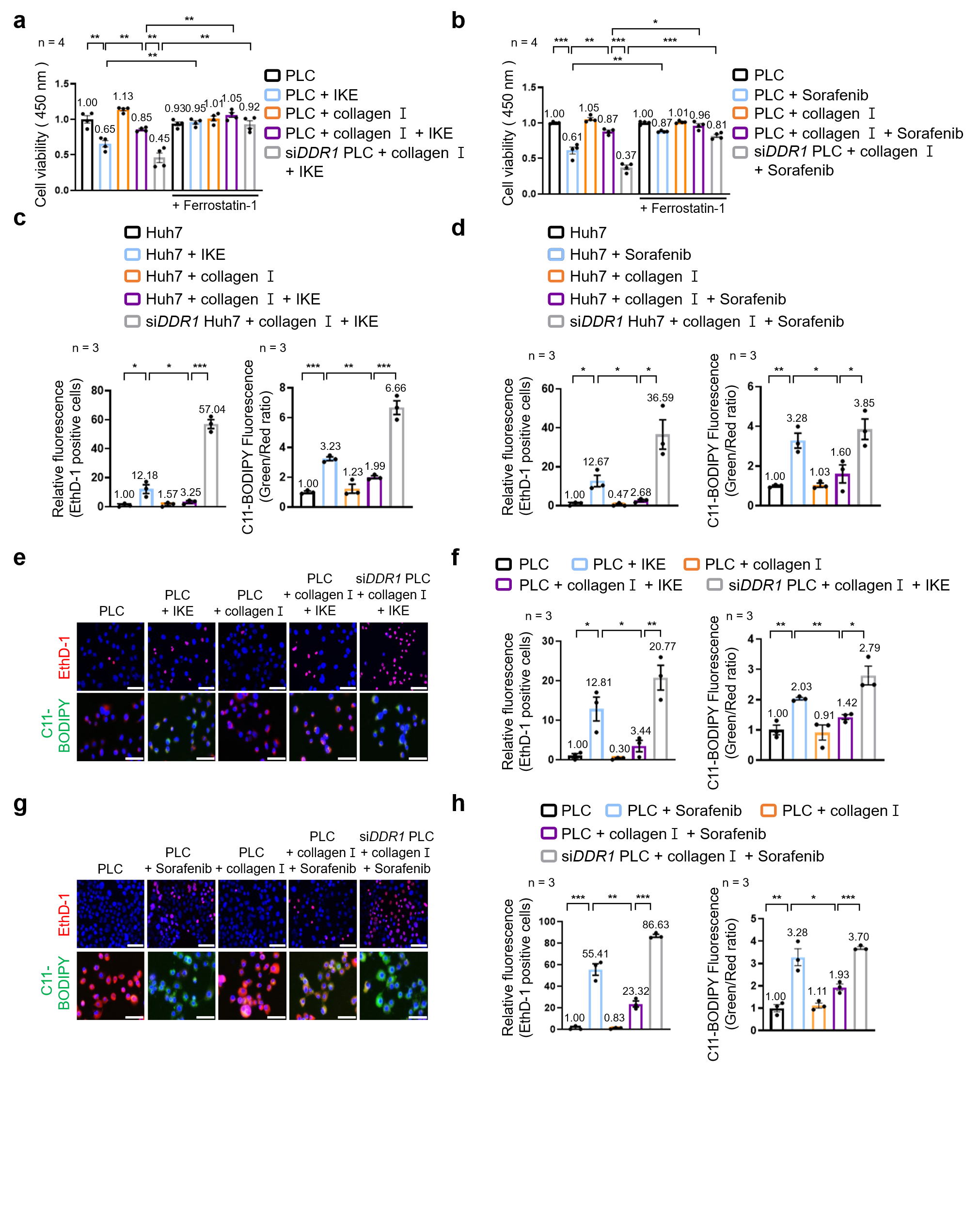
**Supplementary Figure 6 (Related to Fig. 5). Collagen-induced DDR1 signaling in the regulation of ferroptotic cell death in HCC.** (a) Cell viability measured by CCK-8 assay in PLC/PRF/5 cells treated with IKE (1 µM, 6 h) in the presence or absence of collagen I (30 µg/mL, 24 h), ferrostatin-1 (1 µM, 24 h), or *DDR1*-targeting siRNA (n = 4). (b) Cell viability measured by CCK-8 assay in PLC/PRF/5 cells treated with sorafenib (10 µM, 6 h) in the presence or absence of collagen I (30 µg/mL, 24 h), ferrostatin-1 (1 µM, 24 h), or *DDR1*-targeting siRNA (n = 4). (c) Quantification of EthD-1 (red, 4 µM) and C11-BODIPY (green, 10 µM) staining in Huh7 cells treated with IKE (1 µM, 6 h) in the presence or absence of collagen I (30 µg/mL, 24 h) or *DDR1*-targeting siRNA, as depicted in Fig. 5c (*n* = 3). (d) Quantification of EthD-1 (red, 4 µM) and C11-BODIPY (green, 10 µM) staining in Huh7 cells treated with sorafenib (10 µM, 6 h) in the presence or absence of collagen I (30 µg/mL, 24 h) or *DDR1*-targeting siRNA, as depicted in Fig. 5d (*n* = 3). (e, f) Representative images (e) and quantification (f) of EthD-1 (red, 4 µM) and C11-BODIPY (green, 10 µM) staining in PLC/PRF/5 cells treated with IKE (1 µM, 6 h) in the presence or absence of collagen I (30 µg/mL, 24 h) or *DDR1*-targeting siRNA (*n* = 3). (g, h) Representative images (g) and quantification (h) of EthD-1 (red, 4 µM) and C11-BODIPY (green, 10 µM) staining in PLC/PRF/5 cells treated with sorafenib (10 µM, 6 h) in the presence or absence of collagen I (30 µg/mL, 24 h) or *DDR1*-targeting siRNA (*n* = 3). Scale bar: 75 µm. Data are presented as the mean ± SEM from at least three independent experiments. **p* < 0.05; ***p* < 0.01; ****p* < 0.001. PLC, PLC/PRF/5.


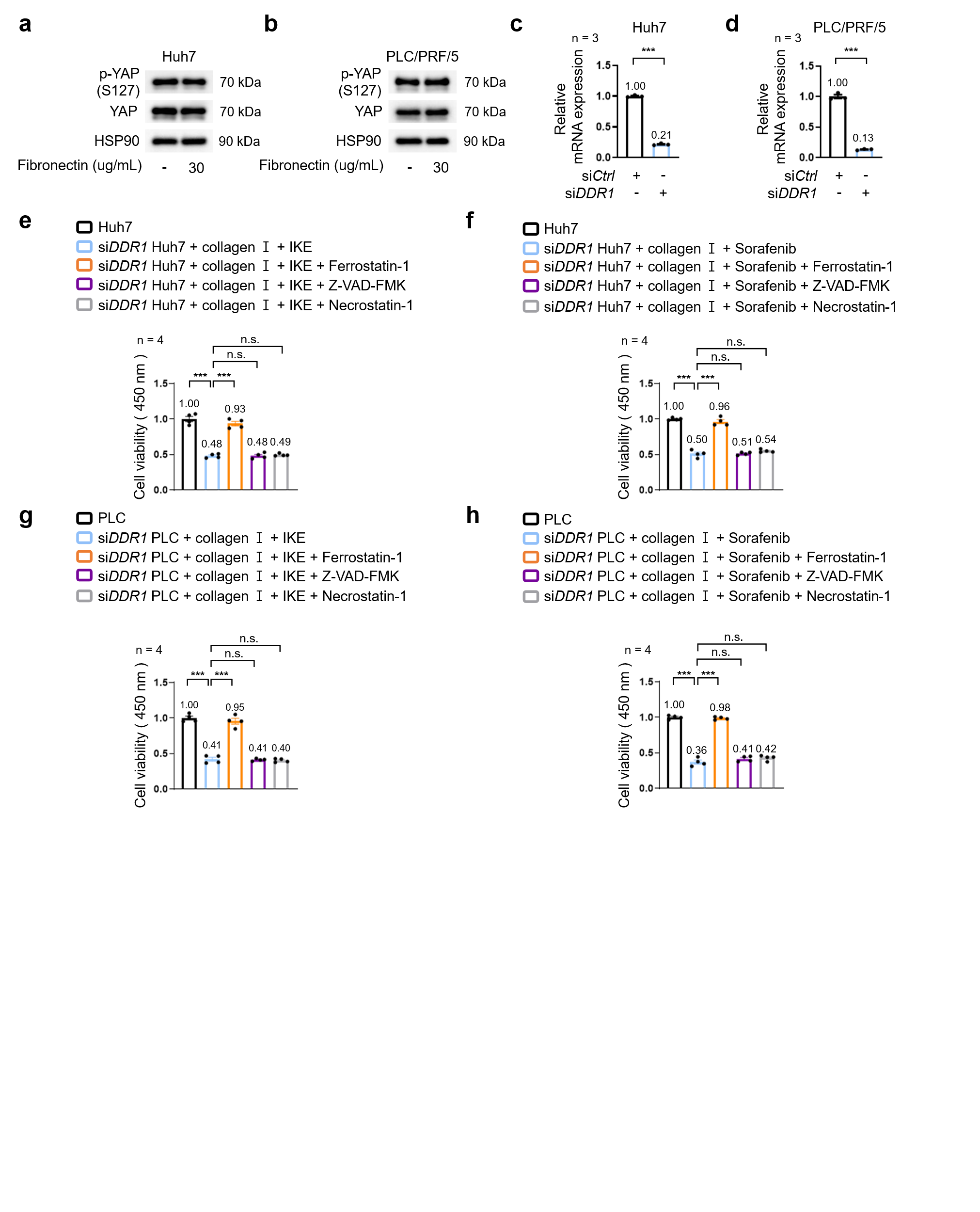
**Supplementary Figure 7 (Related to Fig. 5). Effects of collagen-driven YAP activation on ferroptosis resistance in HCC.** (a, b) Protein levels of phosphorylated YAP (S127) in Huh7 (a) and PLC/PRF/5 (b) cells treated with recombinant human fibronectin (30 µg/mL, 24 h). (c, d) Validation of *DDR1* knockdown efficiency in Huh7 (c) and PLC/PRF/5 (d) cells (n = 3). (e) Cell viability assessed by CCK-8 assay in *DDR1*-silenced Huh7 cells treated with IKE (1 µM, 6 h) in the presence or absence of collagen I (30 µg/mL, 24 h), ferrostatin-1 (1 µM, 24 h), Z-VAD-FMK (20 µM, 24 h), or necrostatin-1 (20 µM, 24 h) (n = 4). (f) Cell viability assessed by CCK-8 assay in *DDR1*-silenced Huh7 cells treated with sorafenib (10 µM, 6 h) in the presence or absence of collagen I (30 µg/mL, 24 h), ferrostatin-1 (1 µM, 24 h), Z-VAD-FMK (20 µM, 24 h), or necrostatin-1 (20 µM, 24 h) (n = 4). (g) Cell viability assessed by CCK-8 assay in *DDR1*-silenced PLC/PRF/5 cells treated with IKE (1 µM, 6 h) in the presence or absence of collagen I (30 µg/mL, 24 h), ferrostatin-1 (1 µM, 24 h), Z-VAD-FMK (20 µM, 24 h), or necrostatin-1 (20 µM, 24 h) (n = 4). (h) Cell viability assessed by CCK-8 assay in *DDR1*-silenced PLC/PRF/5 cells treated with sorafenib (10 µM, 6 h) in the presence or absence of collagen I (30 µg/mL, 24 h), ferrostatin-1 (1 µM, 24 h), Z-VAD-FMK (20 µM, 24 h), or necrostatin-1 (20 µM, 24 h) (n = 4). Data are presented as the mean ± SEM from at least three independent experiments. n.s., not significant; ****p* < 0.001. PLC, PLC/PRF/5.


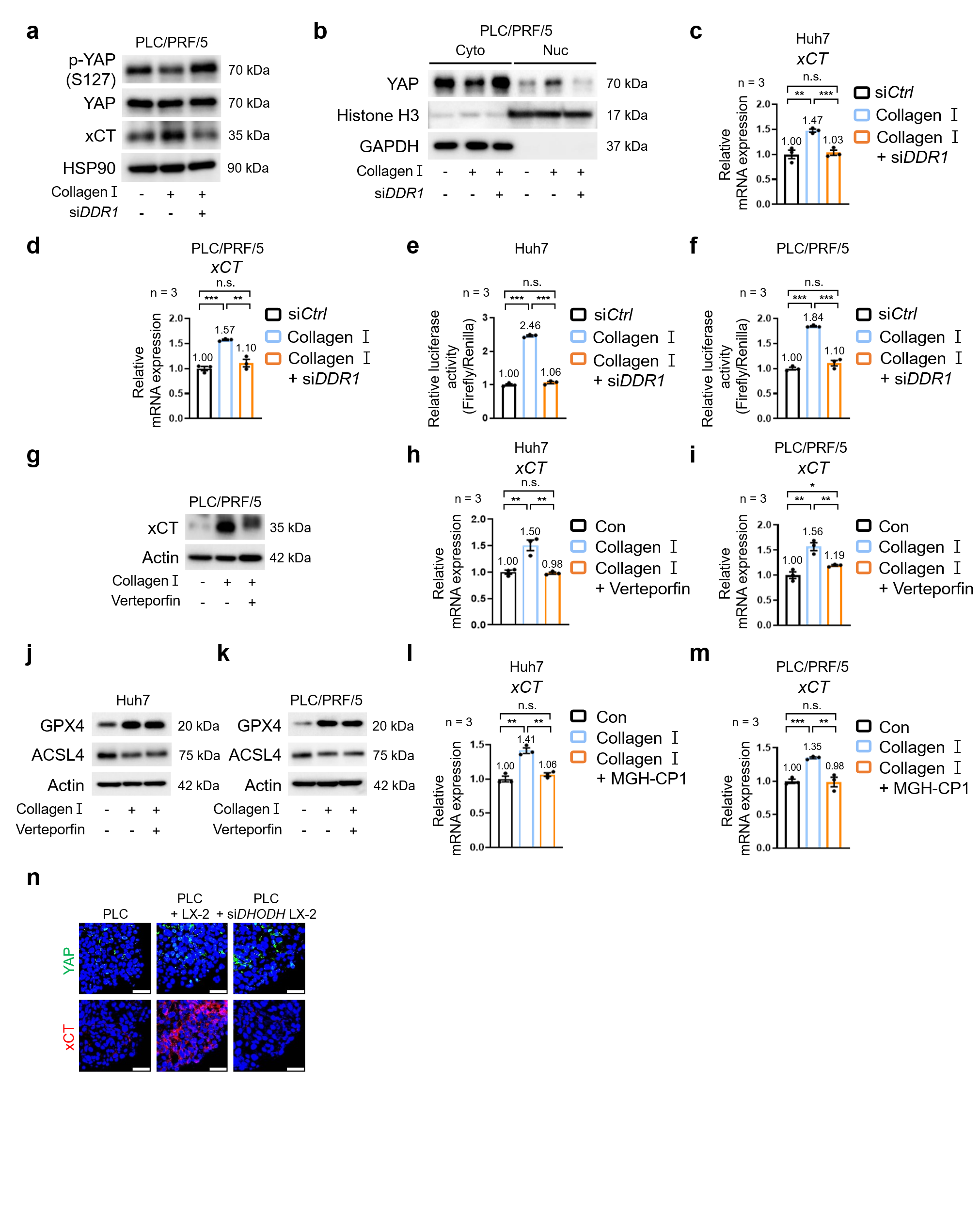
**Supplementary Figure 8 (Related to Fig. 5). Effects of collagen-induced YAP signaling on xCT upregulation and ferroptosis resistance in HCC.** (a) Protein levels of phosphorylated YAP (S127) and xCT in *DDR1*-silenced PLC/PRF/5 cells cultured in the presence or absence of collagen I (30 µg/mL, 24 h). (b) Protein levels of YAP in cytoplasmic and nuclear fractions of *DDR1*-silenced PLC/PRF/5 cells in the presence or absence of collagen I (30 µg/mL, 24 h). Histone H3 and GAPDH were used as nuclear and cytoplasmic markers, respectively. (c, d) Relative *xCT* mRNA expression in *DDR1*-silenced Huh7 (c) and PLC/PRF/5 (d) cells in the presence or absence of collagen I (30 µg/mL, 24 h) (n = 3). (e, f) Relative TEAD luciferase activity in *DDR1*-silenced Huh7 (e) and PLC/PRF/5 (f) cells in the presence or absence of collagen I (30 µg/mL, 24 h) (n = 3). (g) Protein levels of xCT in PLC/PRF/5 cells treated with collagen I (30 µg/mL) or verteporfin (1 µM, 24 h). (h, i) Relative *xCT* mRNA expression in Huh7 (h) and PLC/PRF/5 (i) cells treated with collagen I (30 µg/mL, 24 h) or verteporfin (1 µM, 24 h) (n = 3). (j, k) Protein levels of GPX4 and ACSL4 in Huh7 (j) and PLC/PRF/5 (k) cells treated with collagen I (30 µg/mL) or verteporfin (1 µM, 24 h). (l, m) Relative *xCT* mRNA expression in Huh7 (l) and PLC/PRF/5 (m) cells treated with collagen I (30 µg/mL, 24 h) or MGH-CP1 (2 µM, 24 h) (n = 3). (n) Representative immunofluorescence images of YAP and xCT in *DHODH*-silenced LX-2 cells co-cultured with PLC/PRF/5 cells. Scale bar: 75 µm. Data are presented as the mean ± SEM from at least three independent experiments. n.s., not significant; *p < 0.05; ***p* < 0.01; and ****p* < 0.001. PLC, PLC/PRF/5.


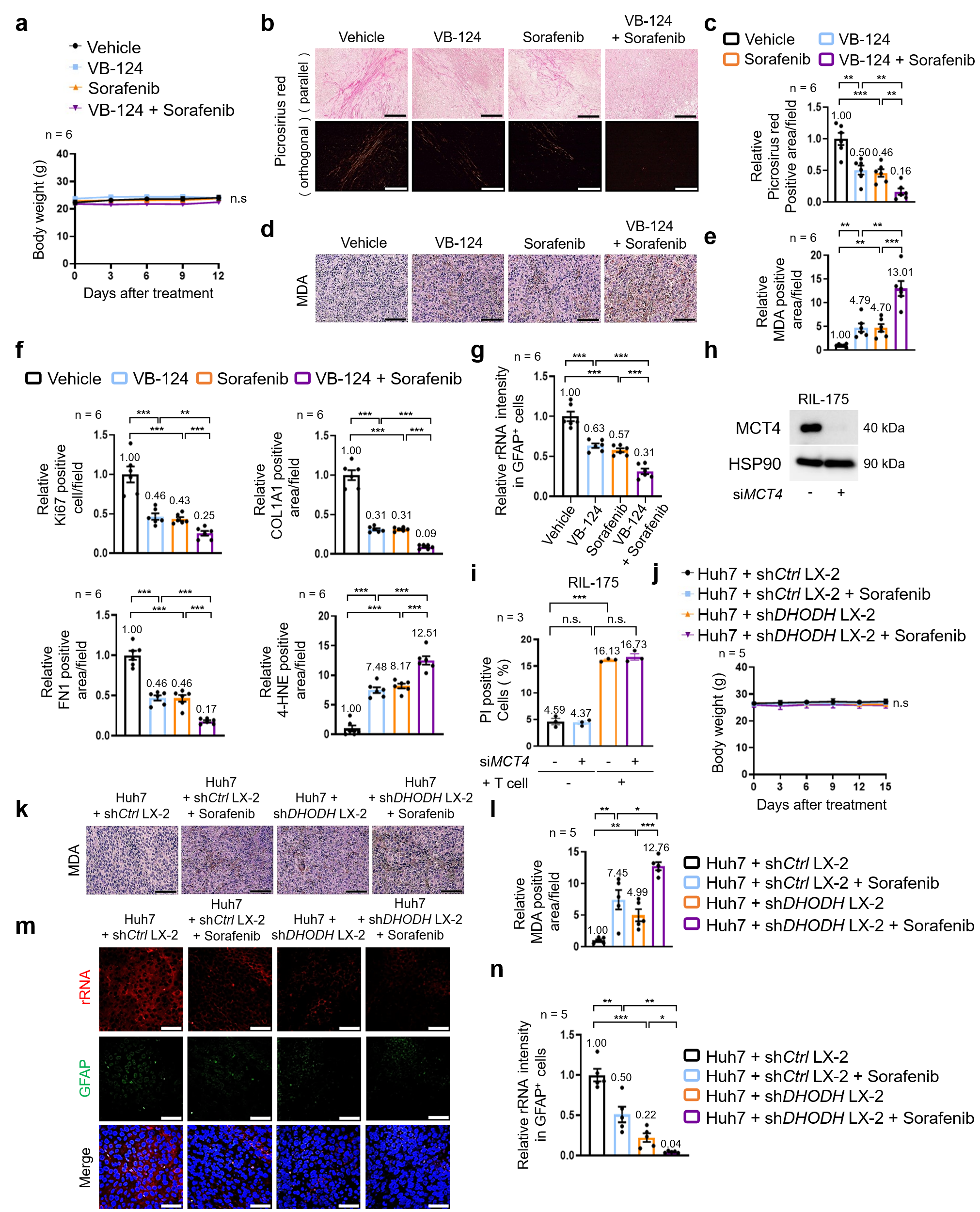
**Supplementary Figure 9 (Related to Fig. 6). Effects of targeting lactate efflux from HCC cells or DHODH in HSCs on rRNA synthesis and ECM deposition *in vivo*.** (a) Body weight of orthotopic RIL-175 tumors from C57BL/6 mice and treated with VB-124 (10 mg/kg) or sorafenib (5 mg/kg) (n = 6 per group). (b, c) Immunohistochemical staining (b) and quantification (c) of picrosirius red (viewed under parallel or orthogonal polarizing filter) in RIL-175 orthotopic tumor tissues (n = 6 per group). (d, e) Immunohistochemical staining (d) and quantification (e) of MDA in RIL-175 orthotopic tumor tissues (n = 6 per group). (f, g) Quantification of orthotopic RIL-175 tumors in C57BL/6 mice treated with VB-124 (10 mg/kg) or sorafenib (5 mg/kg), corresponding to Figure 6d (f) and Figure 6e (g) (n = 6 per group). (h) MCT4 protein levels in *MCT4*-knockdown RIL-175 cells. (i) Percentage of PI-positive in *MCT4*-silenced RIL-175 cells co-cultured with T cells. (j) Body weight of nude mice (n = 5 per group) harboring Huh7 cells mixed with *DHODH*-knockdown LX-2 cells and treated with sorafenib. (k, l) Immunohistochemical staining (k) and quantification (l) of MDA in tumor tissues from nude mice (*n* = 5 per group). (m, n) Representative immunofluorescence images (m) and quantification (n) of rRNA and GFAP staining in tumor tissues from nude mice (n = 5 per group) harboring Huh7 cells mixed with *DHODH*-knockdown LX-2 cells and treated with sorafenib. Scale bar: 75 µm. Data are presented as mean ± SEM from independent mice. n.s., not significant; **p* < 0.05; ***p* < 0.01; ****p* < 0.001.

| **Supplementary Table S1**. Clinical characteristics of patients who underwent resection of HCC | |
| --- | --- |
| Variables |  |
| No. of patients | 206 |
| Sex (male/female) | 154/52 |
| Mean ± SEM age | 56.85 ± 11.26 |
| Cause of HCC |  |
| HBV | 156 |
| HCV | 17 |
| Alcohol | 22 |
| HBV + Alcohol | 3 |
| Cryptogenic | 8 |
| Tumor size (cm) |  |
| <5 | 128 |
| ≥5 | 78 |
| TNM stage |  |
| I–II | 149 |
| III–IV | 57 |

| **Supplementary Table S2.** **List of** **real-time PCR primers** | | | | |
| --- | --- | --- | --- | --- |
| Gene | Forward (5′ → 3′) | | Reverse (3′ → 5′) | |
| Human COL1A1 | ACCCCACTCAGCCCAGTGT | | ACCAGACATGCCTCTTGTCCTT | |
| Human FN1 | CATGAGACTGGTGGTTACATGTTAGA | | GCATGATCAAAACACTTCTCAGCTA | |
| Human LDHA | AGGCTCCCCAGAACAAGATT | | TCTCGCCCTTGAGTTTGTCT | |
| Human MCT4 | GGTGGCTGCGTCCTTTTG | | AACCCAACCCCGTGATGAC | |
| Human RICTOR | AATGCTTCCTTGTTTCCTAGTTCTG | | TGCTCGGACCATTCTGTCTCT | |
| Human RPTOR | TGACCCCAGTGCGAGACA | | AGCACGGATGTTTCCATAGGA | |
| Human MCT1 | GGCAGGCAGCCCTGTGT | | AAAGATACCGAAGAAAACCTGATTGA | |
| Human LDHB | GGCTGAAAAACTTGGCATTCA | | TCCCCCAAAATCCATCCA | |
| Human S6K | GGCCAGCACAGCAAATCC | | TCATTGTCACATCCATCTGCTCTA | |
| Human CAD | CCTTCCCCTTCGTTTCCAA | | CATTAGCCCCACAGGTTCCA | |
| Human 5’ETS | CCTTCCCCAGGCGTCCCTCG | | GGCAGCGCTACCATAACGGA | |
| Human DDR1 | GAACGGAGGGTGTTGGAAGA | | CCTGGGCGGTTGTTGATG | |
| Human DHODH | CCTCACCAGCCAGGATAAGG | | CCCATCGATGCCCAACTCT | |
| Human GPR81 | GCCCTGTGTGGTTTCTGCTT | | AGCCACGGCCAAATTGAA | |
| Human SLC7A11 | ATCTCCCCCAAGGGCATACT | | CACAGGCAGACCAGAAAACCA | |
| Human CD206 | AGGGCAACACCACTTTAAATTCTT | | GCATACGGCCACCACTGACT | |
| Human CD163 | TGCAGAAAACCCCACAAAAAG | | CAAGGATCCCGACTGCAATAA | |
| Human EP300 | TTGAATGTACAGAGTGCGGAAGA | | AACAGCCATCACAGACGAATCC | |
| Human eIF4E | GCCTGGCTGTGACTACTCACTTT | | ATCGTCCTCCCCGTTTGTTT | |
| Human 36B4 | TCGAACACCTGCTGGATGAC | | CCACGCTGCTGAACATGCT | |
| Mouse Col1a1 | GGGCGAGTGCTGTGCTTT | | CCCTCGACTCCTACATCTTCTGA | |
| Mouse Fn1 | GATATCACCGCCAACTCATTCA | | CAGAATGCTCGGCGTGATG | |
| Mouse Cygb | GAGGTGATCGCCGAGGAATT | | TAGATGAGACCCCGCAGCTT | |
| Mouse Gfap | GCTGGAGGGCGAAGAAAAC | | CTTTTGCCCCCTCGGATCT | |
| Mouse Albumin | CGTGTGTTGCCGATGAGTCT | TGGCACACAACTTATCTCCAAAA | |  |
| Mouse 36B4 | ACCTCCTTCTTCCAGGCTTT | CTCCAGTCTTTATCAGCTGC | |  |
